# Histone H3.3 Is Required to Maintain Replication Fork Progression after UV Damage

**DOI:** 10.1016/j.cub.2014.07.077

**Published:** 2014-09-22

**Authors:** Alexander Frey, Tamar Listovsky, Guillaume Guilbaud, Peter Sarkies, Julian E. Sale

**Affiliations:** 1 Medical Research Council Laboratory of Molecular Biology, Francis Crick Avenue, Cambridge CB2 0QH, UK

## Abstract

Unlike histone H3, which is present only in S phase, the variant histone H3.3 is expressed throughout the cell cycle [1] and is incorporated into chromatin independent of replication [2]. Recently, H3.3 has been implicated in the cellular response to ultraviolet (UV) light [3]. Here, we show that chicken DT40 cells completely lacking H3.3 are hypersensitive to UV light, a defect that epistasis analysis suggests may result from less-effective nucleotide excision repair. Unexpectedly, H3.3-deficient cells also exhibit a substantial defect in maintaining replication fork progression on UV-damaged DNA, which is independent of nucleotide excision repair, demonstrating a clear requirement for H3.3 during S phase. Both the UV hypersensitivity and replication fork slowing are reversed by expression of H3.3 and require the specific residues in the α2 helix that are responsible for H3.3 binding its dedicated chaperones. However, expression of an H3.3 mutant in which serine 31 is replaced with alanine, the equivalent residue in H3.2, restores normal fork progression but not UV resistance, suggesting that H3.3[S31A] may be incorporated at UV-damaged forks but is unable to help cells tolerate UV lesions. Similar behavior was observed with expression of H3.3 carrying mutations at K27 and G34, which have been reported in pediatric brain cancers. We speculate that incorporation of H3.3 during replication may mark sites of lesion bypass and, possibly through an as-yet-unidentified function of the N-terminal tail, facilitate subsequent processing of the damage.

## Highlights

• We report a vertebrate cell line completely lacking the histone variant H3.3
• H3.3-deficient cells are hypersensitive to DNA damage
• A supply of H3.3 is required to maintain fork progression after UV damage
• This S phase role requires the distinct chaperone-binding patch of H3.3

## Teaser

Frey et al. describe a vertebrate cell line completely lacking histone variant H3.3 and show that a supply of this histone variant is required to maintain processive replication after exposure to UV light and cellular resistance to DNA damage.

## Results and Discussion

### H3.3-Deficient DT40 Cells Are Viable but Exhibit Alterations in Gene Expression

H3.3 is incorporated throughout the cell cycle [2, 4], particularly in regions of the genome in which histones need to be displaced, such as transcribed genes or regulatory elements [5, 6]. Incorporation in these contexts depends on the histone chaperone HIRA [7] and helps maintain chromatin structure by filling gaps left by loss of H3.1/H4 [5, 8]. H3.3 deposition at transcriptionally active loci has also been proposed to help maintain active expression, possibly by creating a more accessible chromatin structure [2, 9]. However, H3.3 is also incorporated in some repressed loci and at telomeres and pericentric heterochromatin, where deposition depends on the ATRX-DAXX chaperone complex [10, 11, 12]. Although H3.3 is not essential for transcription in *Drosophila*, its loss results in significantly decreased fertility and reduced viability during embryogenesis [13]. Mouse embryonic stem cells with no H3.3B and depleted of H3.3A exhibit altered regulation of polycomb-dependent gene expression that interferes with their ability to differentiate [14]. Mice lacking H3.3B exhibit a semilethal phenotype with reduced growth, anaphase bridging, and karyotypic abnormalities [15]. Recently, H3.3 has also been implicated in the response to ultraviolet (UV) irradiation, because its chaperone HIRA is required to promote transcription restart after UV damage [3].

**Figure 1:**
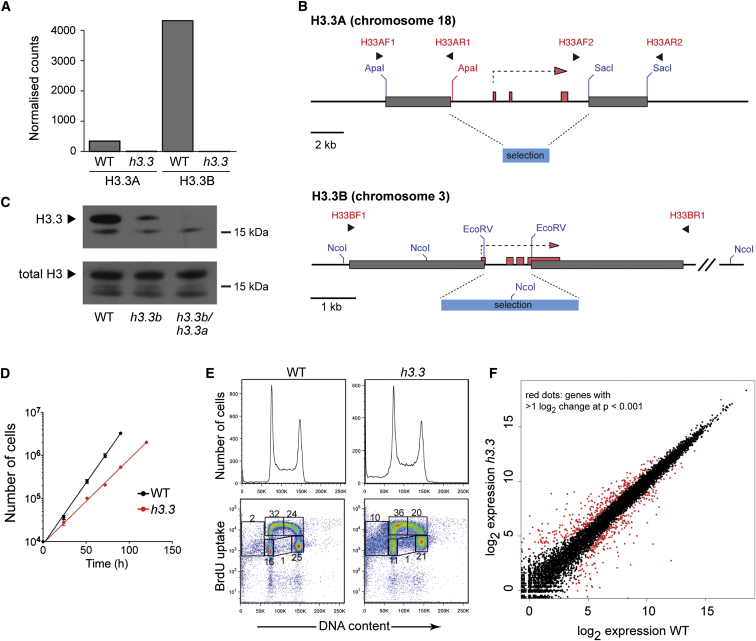
DT40 Cells Deficient in H3.3 (A) Expression of H3.3 from the two alleles H3.3A and H3.3B, monitored by RNA-seq. The y axis represents the normalized number of reads from each locus. (B) Gene targeting strategies for the *H3.3A* and *H3.3B* loci. Exons are shown as salmon pink boxes. The targeting arms are shown as gray boxes and the selection cassette as a blue box. Primers are indicated in red and key restriction sites are in blue (endogenous) or red (introduced during cloning). See also Supplemental Experimental Procedures. (C) Confirmation of loss of H3.3 expression. Western blot of acid-extracted histones for H3.3 and total H3 from wild-type, cells lacking H3.3B (*h3.3b*), and cells lacking both H3.3A and H3.3B (*h3.3b/h3.3a*; abbreviated *h3.3*). (D) Growth of wild-type and *h3.3* cells. Each point represents the average cell number for three experiments with error bars showing 1 SD. The lines are a linear regression fit. (E) One- and two-dimensional cell-cycle analysis of asynchronous populations of wild-type and *h3.3* DT40. Each plot shows a total of 50,000 cells, and the percentage of the total cycling cells in each gate is indicated. BrdU, bromodeoxyuridine. (F) RNA-seq from wild-type versus *h3.3*. The log_2_ expression level for each gene is determined from the normalized counts of three wild-type and three H3.3 RNA-seq experiments. Red dots represent genes whose expression differs by greater than 2-fold with p < 0.001. See also Figure S1 and Table S1.

In order to examine the effect of complete loss of H3.3 in a differentiated vertebrate cell line, we created an H3.3 null variant of the chicken bursal lymphoma DT40 [16]. In chicken, as in mammals, H3.3 is encoded by two loci, *H3.3A* on chromosome 18 and *H3.3B* on chromosome 3. Despite considerable divergence of the cDNA sequence of H3.3A and H3.3B, they encode identical proteins, which also have the same sequence as human H3.3. RNA deep-sequencing analysis (RNA-seq) of DT40 revealed that H3.3B contributes over 90% of the total pool of H3.3 transcript in chicken DT40 B cells (Figure 1A).

To create H3.3 null DT40 cells, we first disrupted both alleles of *H3.3B* by homologous recombination using a targeting strategy that removed the majority of the coding sequence (Figure 1B; Supplemental Experimental Procedures available online). This resulted in a substantial reduction of total H3.3 protein levels (Figure 1C), as predicted by the RNA-seq data (Figure 1A). We then disrupted both alleles of *H3.3A* by removing the whole H3.3A coding sequence (Figure 1B). This resulted in loss of the remaining H3.3 protein (Figure 1C). We subsequently refer to this H3.3 null line as *h3.3* DT40. *h3.3* cells proliferate more slowly than wild-type (c. 15 versus c. 11 hr; Figure 1D). Their unperturbed cell-cycle profile suggests that this is at least in part explained by an increase in spontaneous apoptosis (Figure 1E).

We next examined the extent of transcriptional dysregulation in cells lacking H3.3 by RNA-seq. This analysis revealed that 557 of 16,396 gene transcripts (3.4%) exhibited a >2-fold and significant (p < 0.001) change in expression (Figure 1F; Table S1). Interestingly, the number of genes exhibiting a significant decrease in expression (235) is actually slightly exceeded by those increasing in expression (324), supporting recent evidence that H3.3, or its modifications, is not just important for actively expressed loci [12, 14]. We observed no underlying pattern to the chromosomal locations of affected genes (Figure S1). Thus, loss of H3.3 is linked to significant changes in gene expression, but affects a relatively small fraction of loci in DT40 cells.

### H3.3 Is Likely to Operate in Concert with the Nucleotide Excision Repair Pathway

**Figure 2:**
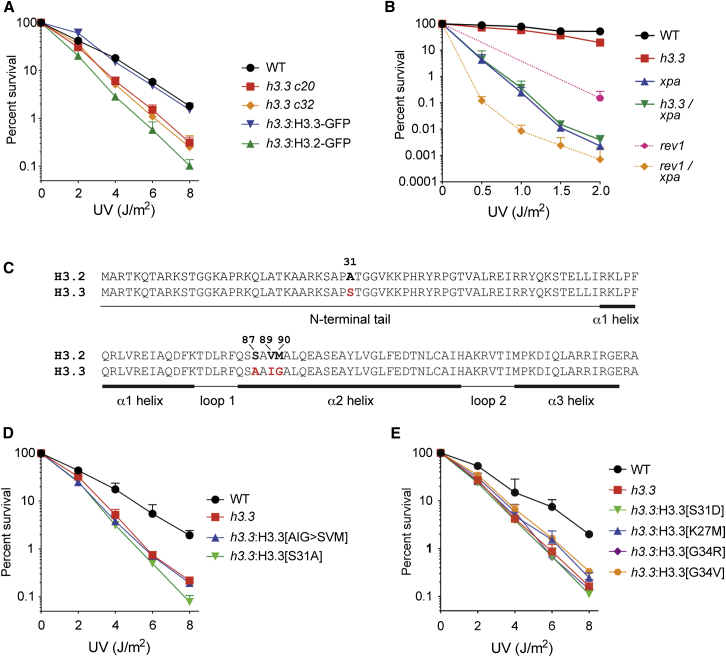
DNA Damage Sensitivity of H3.3-Deficient Cells Colony survival assays following exposure to UV light. (A) Complementation of the DNA damage sensitivity of *h3.3* cells with H3.3 and H3.2. Two clones of the *h3.3* knockout (c20 and c32) are shown. Fold sensitivities versus wild-type: *h3.3 c20* 1.4; *h3.3 c32* 1.4; *h3.3*:H3.3-GFP 1; *h3.3*:H3.2-GFP 1.7. (B) Epistasis of XPA to H3.3. That the colony survival assay has the power to detect additional sensitivity beyond that of the *xpa* mutant is demonstrated by a *rev1/xpa* mutant, which lacks both NER and tolerance of UV lesions during replication by translesion synthesis. Fold sensitivities versus wild-type: *h3.3* 2.1; *xpa* 17.5; *h3.3/xpa* 16.7; *rev1* 9; *rev1/xpa* 310. (C) Alignment of chicken H3.2 and H3.3. The key differences are highlighted and the domain structure of the protein is indicated below the alignment. (D) Complementation of *h3.3* cells with H3.3 with a mutated chaperone-binding patch (abbreviated AIG>SVM) or S31A. Fold sensitivities versus wild-type: *h3.3* 1.6; *h3.3*:H3.3[AIG>SVM] 1.7; *h3.3*:H3.3[S31A] 1.8. (E) Effect of expression of H3.3 carrying a potentially phosphomimetic mutation of S31, S31D, or three nearby cancer-associated mutations, H3.3[K27M], H3.3[G34R], and H3.3[G34V]. Fold sensitivities versus wild-type: *h3.3* 1.7; *h3.3*:H3.3[S31D] 1.8; *h3.3*:H3.3[K27M] 1.6; *h3.3*:H3.3[G34R] 1.6; *h3.3*:H3.3[G34V] 1.5. Survival assays were performed three times and 1 SD of the surviving fraction is indicated. For clarity, only the positive error bar is shown. See also Figure S2.

In addition to being incorporated during transcription, recent experiments have shown that H3.3 is deposited at sites of UV-induced DNA damage by the histone chaperone HIRA, where it facilitates the recovery of transcription after repair of the damage [3]. We therefore asked whether *h3.3* cells exhibit sensitivity to UV light. *h3.3* cells were modestly, but consistently, hypersensitive to UV irradiation (Figure 2A). This is unlikely to be a secondary effect, because no known DNA damage response genes exhibited significantly dysregulated expression in *h3.3* cells (Table S1). Further, the sensitivity of *h3.3* cells to UV light was reversed by stable expression of H3.3 C-terminally tagged with GFP (Figure 2A; Figure S2). H3.2 could not substitute for H3.3 in rescuing the UV sensitivity of *h3.3*. In fact, ectopic expression of H3.2 appeared to cause further sensitization to UV, as previously observed in yeast [17]. Because H3.3 has been implicated in processes related to nucleotide excision repair (NER) [3], we examined its genetic relationship to NER by performing epistasis analysis of H3.3 with XPA, a key component of the NER pathway. *xpa* DT40 cells are highly sensitive to UV light, considerably more so than *h3.3* (Figure 2B). A double *h3.3/xpa* mutant was no more sensitive than *xpa* alone, suggesting that XPA may be epistatic to H3.3 and that H3.3 acts to facilitate excision repair of a subset of UV lesions. However, although the UV colony survival assay has the dynamic range to detect additional sensitivity over and above that of the *xpa* mutant (Figure 2B), the very large difference in the sensitivities of the *h3.3* and *xpa* mutants means that epistasis in this instance must be interpreted with some caution.

### Resistance to DNA Damage Requires the H3.3-Specific Chaperone-Binding Patch and S31

H3.3 differs at two sites from H3.2, the single canonical H3 in chickens (Figure 2C). S31, in the N-terminal tail region and an alanine in H3.2, has been reported to be phosphorylated during mitosis, although the function of this modification is not yet understood [18]. H3.3 also has three residues at the base of α helix 2 that differ from H3.2. These are A87/I89/G90, which are S87/V89/M90 in H3.2 (hereafter referred to as “AIG” and “SVM”). This “patch” is thought to define the chaperone specificity of H3.2 and H3.3. Thus, the AIG patch is required for binding of H3.3 to DAXX [10] and is required for replication-independent chromatin deposition [2, 4], that is dependent on HIRA [7]. We created *h3.3* clones stably expressing H3.3-GFP carrying either a substitution of the AIG patch with the SVM patch of H3.3 or an S31A substitution and ensured matched expression levels by monitoring GFP by flow cytometry (Figure S2). Neither the AIG patch nor S31A H3.3 mutants complemented the UV hypersensitivity of *h3.3* cells (Figure 2D), suggesting that the chaperone binding specificity of H3.3 and a serine at position 31 are required. A potentially phosphomimetic substitution of S31 with aspartic acid also did not complement the UV sensitivity of *h3.3* cells (H3.3[S31D]; Figure 2E).

### Pediatric Cancer-Associated H3.3 Mutations near S31 Also Result in UV Sensitivity

Recently, mutations in the N-terminal tail of H3.3, in the vicinity of S31, have been linked to a number of pediatric cancers, including glioblastoma, chondroblastoma, and giant cell tumors of bone [19, 20, 21]. Understanding the mechanistic basis for the clinical effects of these apparently driver mutations has focused on their effects on posttranslational modifications of H3. Thus, mutations at G34 affect the global distribution of H3K36me3 and changes in gene expression [22]. Likewise, mutation of H3.3K27, a residue whose trimethylation is associated with polycomb complex-mediated transcriptional repression, results in reduced global H3K27me3 and derepression of multiple transcripts [23]. Because S31 lies close to these residues, we wondered whether the cancer-associated mutations K27M, G34R, and G34V [19, 20] might also confer sensitivity to DNA damage. Interestingly, all three H3.3 mutants exhibit UV sensitivity similar to the *h3.3* knockout, suggesting that these residues are also required for the role played by H3.3 in facilitating excision repair (Figure 2E). This somewhat surprising result suggests the possibility that H3.3 cancer-associated mutations could impact on DNA repair as well as on transcriptional regulation, a point that merits further exploration.

### H3.3 Is Required during S Phase to Maintain Processive Replication after UV Irradiation

**Figure 3:**
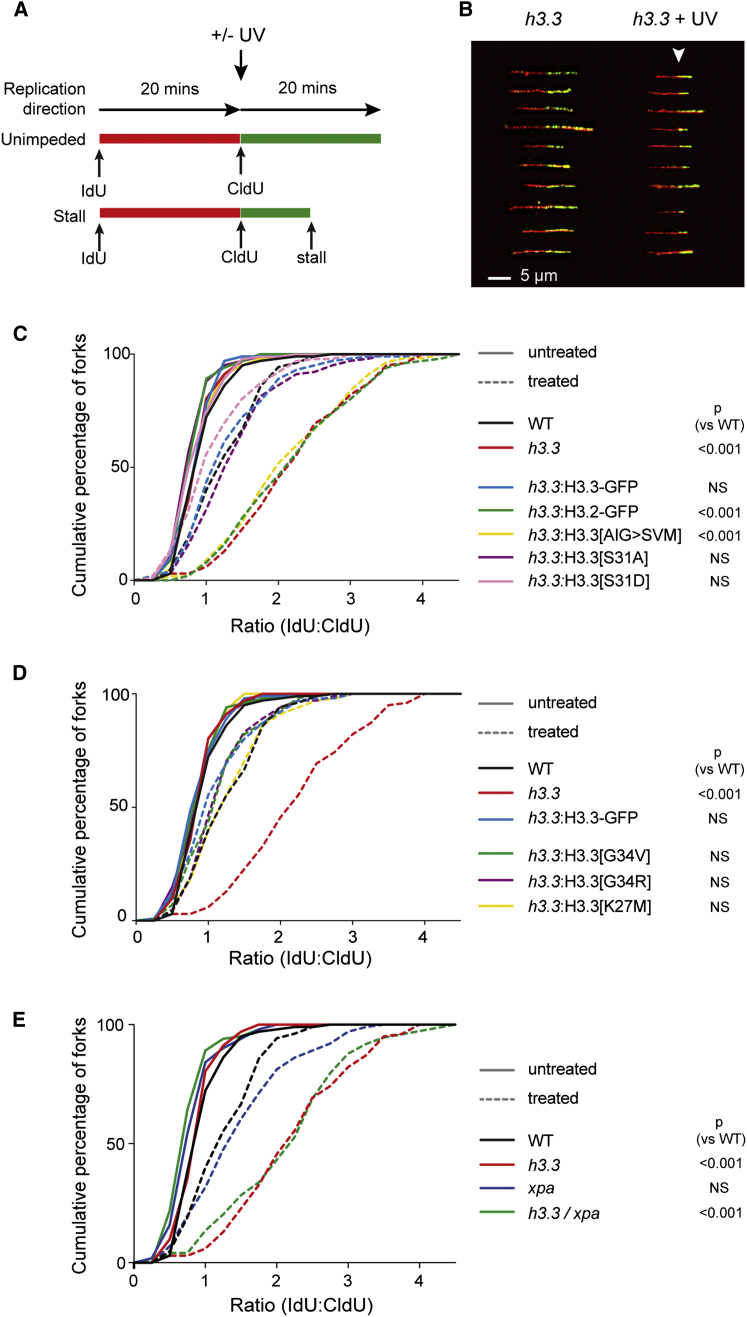
H3.3 Is Required to Maintain Replication Fork Progression after UV Exposure (A) Schematic of the fork labeling experiment. IdU, iododeoxyuridine; CldU, chlorodeoxyuridine. (B) Sample pictures of DNA fibers labeled from *h3.3* cells. The point of UV exposure is indicated by the white arrowhead. (C–E) Replication fork stalling of wild-type or mutants in response to either sham irradiation (solid lines) or 40 J/m^2^ 265 nm UV light (dashed lines). The ratio of the length of the second label to the first is calculated for each fiber, and the data are presented as a cumulative percentage of forks at each IdU:CldU ratio. The p value that the cumulative distribution with UV is different from wild-type is shown (Kolmogorov-Smirnov test). NS, not significant (i.e., p > 0.001). See also Figure S3.

Histone supply affects the processivity of DNA replication [24]. Although the deposition of H3.3 is primarily replication independent, we asked whether the absence of H3.3 affected replication by monitoring fork progression in stretched DNA fibers. We pulse labeled cells sequentially with two different halogenated nucleosides (iododeoxyuridine and chlorodeoxyuridine; 20 min each), stretched the extracted DNA on glass slides, and revealed the replicons with antibodies specific for the halogenated nucleotides (Figure 3A). Loss of H3.3 did not affect replication dynamics in unperturbed conditions. We observed a small, but not significant, decrease in median fork velocity in *h3.3* cells but no change in replication origin density (Figures S3A–S3C). However, after UV irradiation, applied at the same time as the second label, replication fork progression in the second 20 min was dramatically reduced in *h3.3* cells in comparison to wild-type (Figures 3B and 3C). It is likely that at least some of these forks remain persistently blocked, because a greater fraction of *h3.3* cells accumulate in late S phase 24 hr after UV exposure, suggesting a delay in completion of replication (Figure S3D). The delayed fork progression following UV exposure in *h3.3* cells was reduced to wild-type levels by expression of H3.3-GFP but not H3.2-GFP (Figure 3C). Although this defect is reminiscent of cells lacking the translesion polymerase REV1 [25, 26], we could observe robust translesion synthesis of UV (6-4) photoproducts in *xpa/h3.3* cells using a replicating plasmid assay [27] and, further, the frame infidelity characteristic of photoproduct bypass in REV1-deficient cells [27] was not evident (Figures S3E–S3G). Thus, delayed replication fork progression after UV damage in *h3.3* cells does not appear to result from a significant defect in REV1-dependent translesion DNA synthesis. We then asked whether the role of H3.3 at the replication fork was also dependent on both the AIG patch and S31, as for UV sensitivity. Whereas the AIG-to-SVM patch mutant failed to complement the defective fork progression after UV (Figure 3C), the H3.3[S31A] mutant restored wild-type behavior (Figure 3C), as did the cancer-associated mutants G34V, G34R, and K27M (Figure 3D). In view of the apparent epistasis of H3.3 and XPA, we considered whether the delayed fork progression in *h3.3* cells reflected defective excision repair. However, *h3.3* cells exhibit a much more prominent defect in fork progression after UV than *xpa* cells, the response of which is similar to wild-type (Figure 3E). This is not consistent with the fork progression defect seen after UV in *h3.3* cells resulting from defective NER at the fork, an event that in any case would likely be deleterious to cell survival due to strand incision at the lesion causing replication fork collapse.

**Figure 4:**
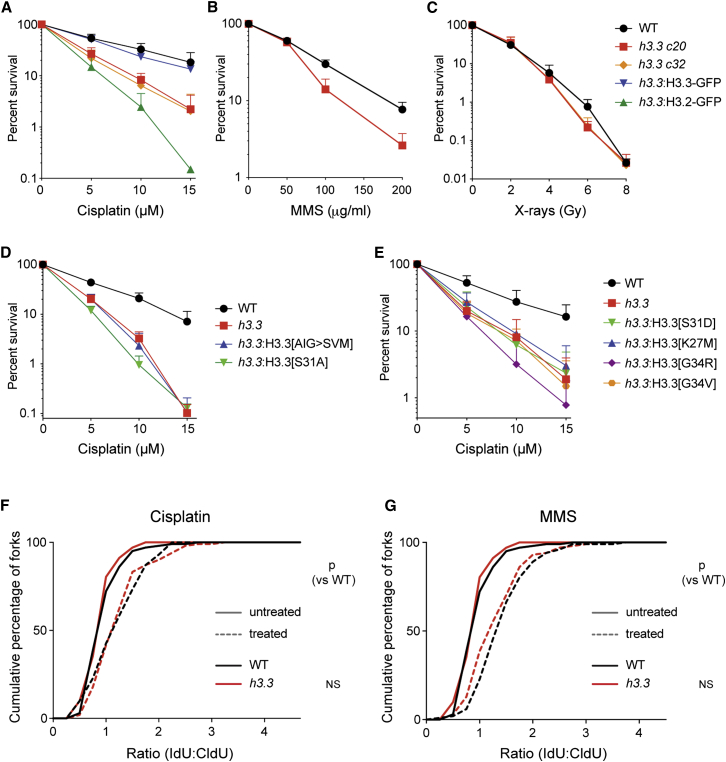
Response of *h3.3* Cells to Other Forms of DNA Damage (A) Sensitivity of *h3.3* cells to cisplatin. Fold sensitivities versus wild-type: *h3.3 c20* 2.5; *h3.3 c32* 2.8; *h3.3*:H3.3-GFP 1.4; *h3.3*:H3.2-GFP 4.1. (B) Sensitivity of *h3.3* cells to methyl methanesulfonate. Fold sensitivity versus wild-type: *h3.3* 1.4. (C) Sensitivity of *h3.3* cells to X-rays. Fold sensitivities versus wild-type: *h3.3 c20* 1.1; *h3.3 c32* 1.1. (D) Sensitivity of *h3.3* AIG patch and S31A mutants to cisplatin. Fold sensitivities versus wild-type: *h3.3* 2.3; *h3.3*:H3.3[AIG>SVM] 2.3; *h3.3*:H3.3[S31A] 2.6. (E) Sensitivity of *h3.3* N-terminal tail mutants to cisplatin. Fold sensitivities versus wild-type: *h3.3* 2.2; *h3.3*:H3.3[S31D] 2.1; *h3.3*:H3.3[K27M] 1.9; *h3.3*:H3.3[G34R] 2.7; *h3.3*:H3.3[G34V] 2.3. Survival assays were performed three times and 1 SD of the surviving fraction is indicated. For clarity, only the positive error bar is shown. (F) Replication fork stalling of wild-type or mutants in response to either sham treatment (solid lines) or 2.5 mM cisplatin (dashed lines). (G) Replication fork stalling of wild-type or mutants in response to either sham treatment (solid lines) or 0.05% methyl methanesulfonate (dashed lines). The p value that the cumulative distribution with UV is different from wild-type is shown (Kolmogorov-Smirnov test). NS, not significant (i.e., p > 0.001).

Finally, we asked whether the role of H3.3 in the response to UV was also seen with other forms of DNA damage. In addition to UV, *h3.3* cells exhibit mild hypersensitivity to the interstrand crosslinking agent cisplatin and the alkylating agent methyl methanesulfonate (MMS) but not to X-rays (Figures 4A–4C). In the case of cisplatin, both the AIG patch and N-terminal tail mutants discussed above exhibit hypersensitivity (Figures 4D and 4E), as observed with UV. However, for neither cisplatin nor MMS is there any exacerbation of the delay in fork progression induced by these agents (Figures 4F and 4G), a point we consider further below.

Our observations provide the first clear evidence of the involvement of a variant histone in replication fork progression, and suggest that forks require a supply of H3.3 when they encounter UV damage to maintain processive replication. Although our experiments are not able to show directly that H3.3 is incorporated by the replication fork during replication of UV DNA damage, by analogy with the effect of histone supply on bulk DNA replication [24], we suggest that the defective fork progression in *h3.3* cells is a result of failure of a process that would normally see H3.3 incorporated. We speculate that H3.3 incorporation during the replication of UV lesions at the fork, and possibly during postreplicative lesion bypass, may facilitate subsequent access and repair (Figure S4). H3.3 incorporation would imply the need for an H3.3-specific chaperone. HIRA would seem to be a strong candidate given its documented role in H3.3 incorporation at sites of UV damage [3], although the same study reported that HeLa cells depleted for HIRA are not UV sensitive [3]. Whether ATRX plays any role in replicating UV-damaged DNA is unknown, but it has been implicated in the replication of G quadruplex DNA [28] and, recently, ATRX-deficient cells have been shown to exhibit replication defects, suggesting that it contributes to limiting fork stalling during S phase [29].

Although cells lacking H3.3 are sensitive to UV, MMS, and cisplatin, a fork progression defect, as assessed in labeled DNA fibers, is only observed after UV exposure. This suggests a broad requirement for H3.3 in facilitating DNA repair, but that incorporation of this histone variant may not only take place “on the fly” at the replication fork when it encounters DNA damage but also, for instance, during lesion bypass in postreplicative gaps. Loss of this latter role would not be detectable as a defect in the DNA fiber assay. The basis for this specificity remains unclear, but we speculate that it may be related to the mechanisms and complexes cells bring into play at different lesions, which in turn may affect the timing of lesion bypass [30]. Indeed, such damage-dependent specificity is now well documented in translesion synthesis [31] and, recently, damage caused by UV and by MMS has been shown to induce quite distinct bypass responses in human cells [32]. However, much further work is needed to understand the contexts in which H3.3 is required for processive replication of damaged DNA.

Finally, how might H3.3 incorporation facilitate subsequent DNA repair and survival? Because it has been proposed that H3.3-containing chromatin has a more open and accessible structure [9], its incorporation may be particularly important for promoting NER in highly condensed regions of the genome. Additionally, the damage sensitivity of the *h3.3* cells may also be related to its ability to promote transcriptional recovery after repair [3], possibly through its ability to interact specifically with components of the FACT chromatin remodeling complex [12], which has itself been implicated in transcriptional recovery after NER and in resistance to UV damage [33].

## Author Contributions

A.F. performed the experiments, analyzed the data, and wrote the paper. T.L. with A.F. performed the UV DNA fiber analysis. G.G. performed the cell-cycle analysis and global analysis of replication by DNA combing and analyzed the RNA-seq data. P.S. created the *h3.3b* line. J.E.S. conceived the study, analyzed the data, and wrote the manuscript.

## Accession Numbers

The ArrayExpress (https://www.ebi.ac.uk/arrayexpress) accession number for the RNA-sequencing data reported in Figure 1 is EMTAB-2754.

## Supplemental Information

Document S1. Supplemental Experimental Procedures and Figures S1–S4

Table S1. List of Genes Significantly Upregulated or Downregulated in *h3.3* versus Wild-Type RNA-Seq, Related to Figure 1

Document S2. Article plus Supplemental Information
