# Supplementary material for: Histone H3.3 Is Required to Maintain Replication Fork Progression after UV Damage
Source: Curr Biol. 2014 Sep 22;24(18):2195–201. doi: 10.1016/j.cub.2014.07.077 (PMC4175177; doi:10.1016/j.cub.2014.07.077)
Supplement: Document S1. Supplemental Experimental Procedures and Figures S1–S4 [file mmc1.pdf]

Current Biology, Volume *24*

Supplemental Information

Histone H3.3 Is Required to Maintain Replication Fork Progression after UV Damage

Alexander Frey, Tamar Listovsky, Guillaume Guilbaud, Peter Sarkies, and Julian E. Sale

Figure S1

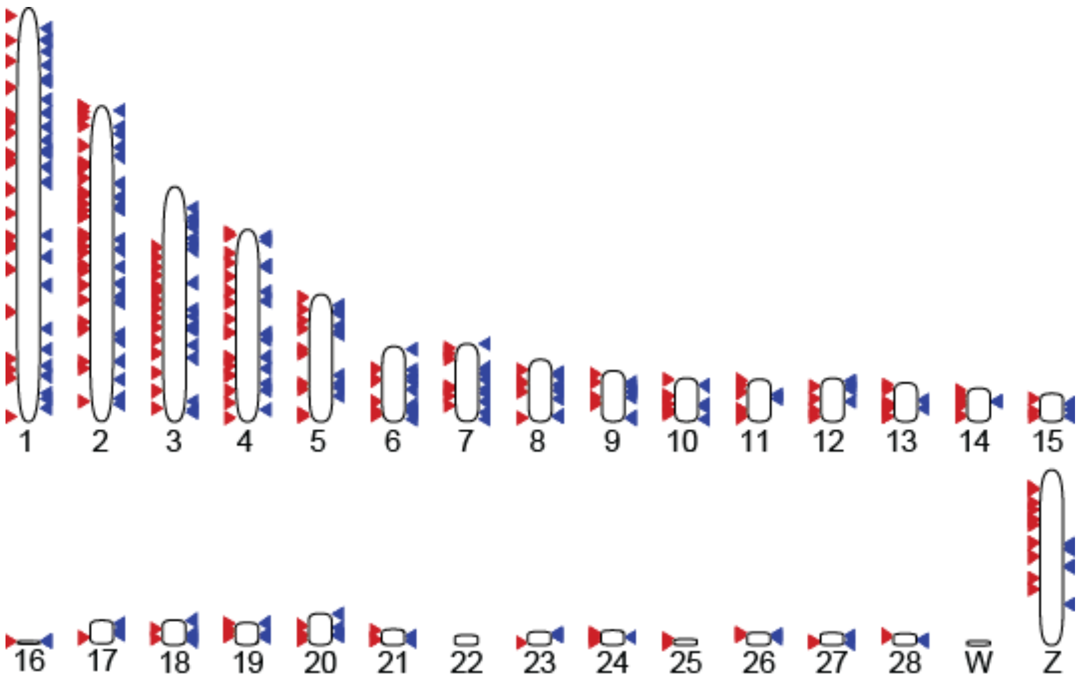

Figure S1. (Related to Figure 1). Dysregulated genes (Table S1) mapped to the genome. Genes with increased expression in *h3.3*, red; genes with decreased expression in *h3.3*, blue.

Figure S2

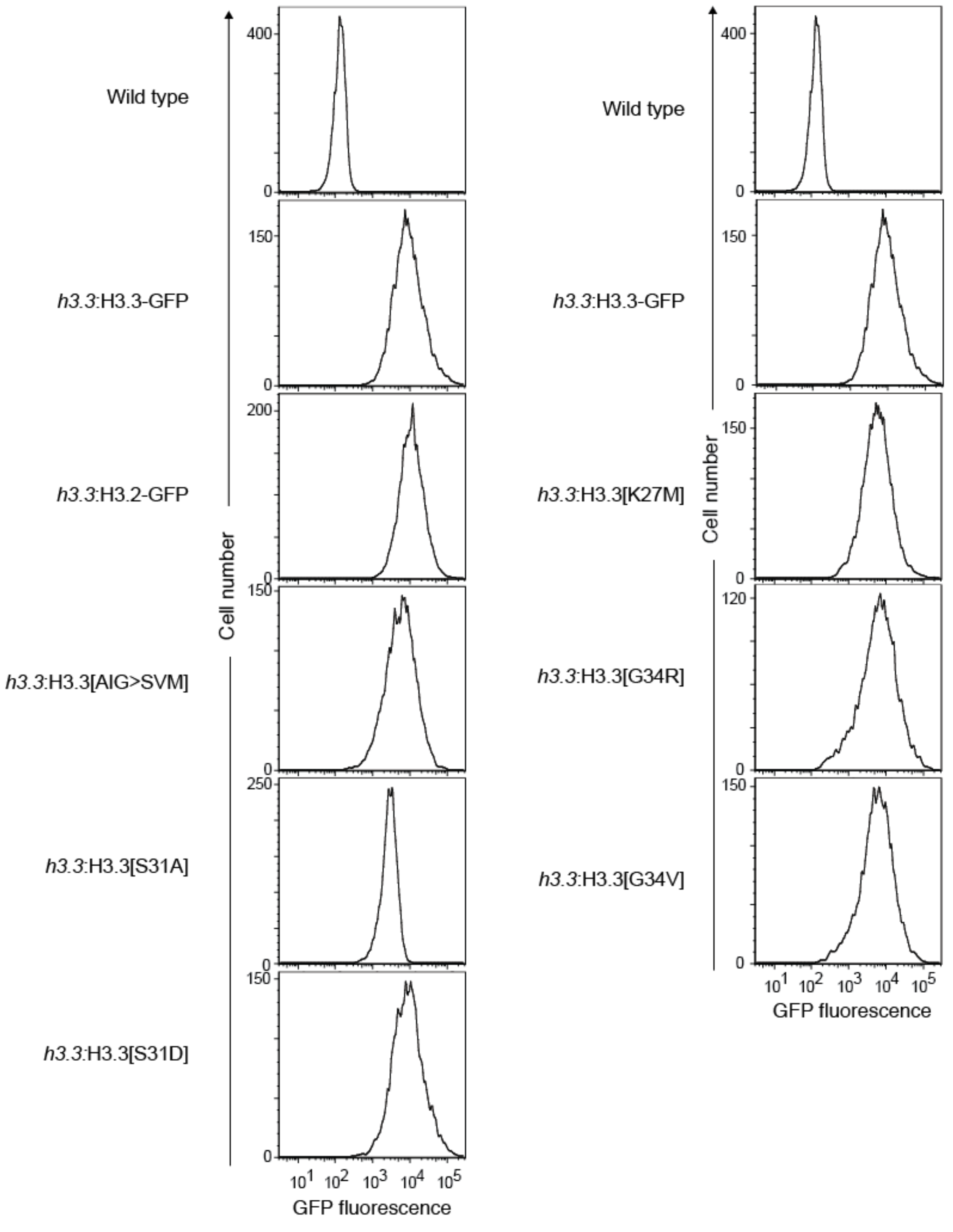

Figure S2. (Related to Figure 2). Flow cytometry for GFP was used to match expression of GFP-histone fusions expressed in the complemented *h3.3* lines used in this study.

Figure S3

A

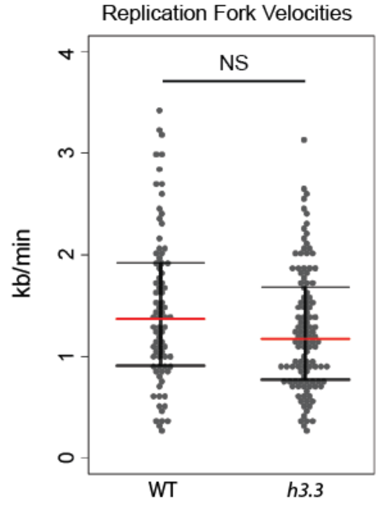

B

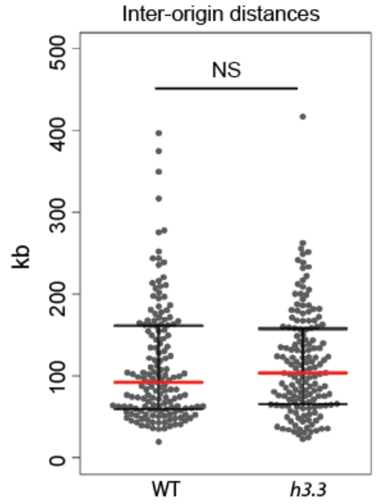

C

	WT	*h3.3*
Mean fibre length (kb)	701 ± 51 (n=65)	535 ± 44 (n=73)
Total DNA analysed (Mb)	38.6	38.2
Origin density (per Mb)	5.4	5.8
Inter-origin distance (kb)	114 ± 6 (n=156)	115 ± 5 (n=156)
Fork velocity (kb/min)	1.48 ± 0.09 (n=79)	1.25 ± 0.06 (n=114)

Figure S3. (Related to Figure 3). A – C. Unperturbed replication dynamics in *h3.3* cells monitored in DNA counterstained fibres (see Supplemental Experimental Procedures). Only the first labelling period (IdU) is measured, as described in reference [S1]. A. Replication fork velocities. B Interorigin distances. In A and B the red line represents the median and whiskers = interquartile range. Mann-Whitney test: p = not significant (NS; $p > 0.05$). C. Summary analysis. Values are presented as mean +/- SEM.

Figure S3 (continued)

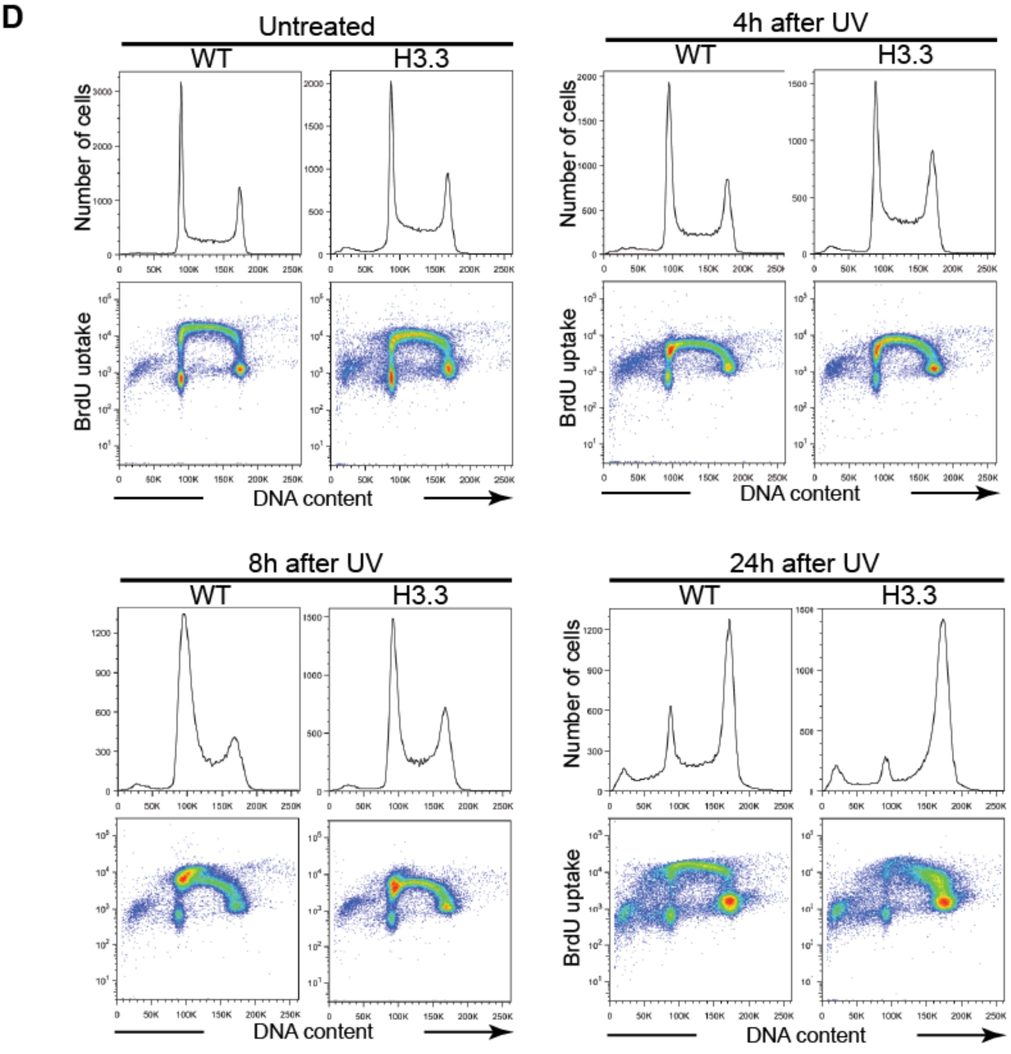

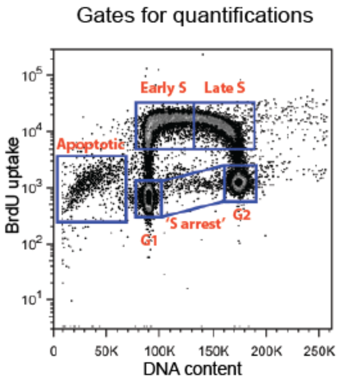

Time after UV

% of cycling cells	Mock WT	Mock *H3.3*	4h WT	4h *H3.3*	8h WT	8h *H3.3*	24h WT	24h *H3.3*
G1	21	17	10	6	6	7	8	4
Early S	38	41	46	46	65	51	25	10
Late S	20	20	22	23	18	23	17	34
G2	19	18	19	22	12	17	44	38
S arrest	1	2	1	1	1	1	4	2
Apoptotic	2	6	7	5	3	4	12	12

Figure S3 (continued). (Related to Figure 3). D. Cell cycle response of wild type and *h3.3* cells to UV irradiation. 1D and 2D cell cycle analysis of asynchronous populations of wild type and *h3.3* cells before and after exposure to 3 Jm^{-2} UVC irradiation. A key for the gating is shown and a table for the percentage of counts in each gate for each condition.

Figure S3 (continued)

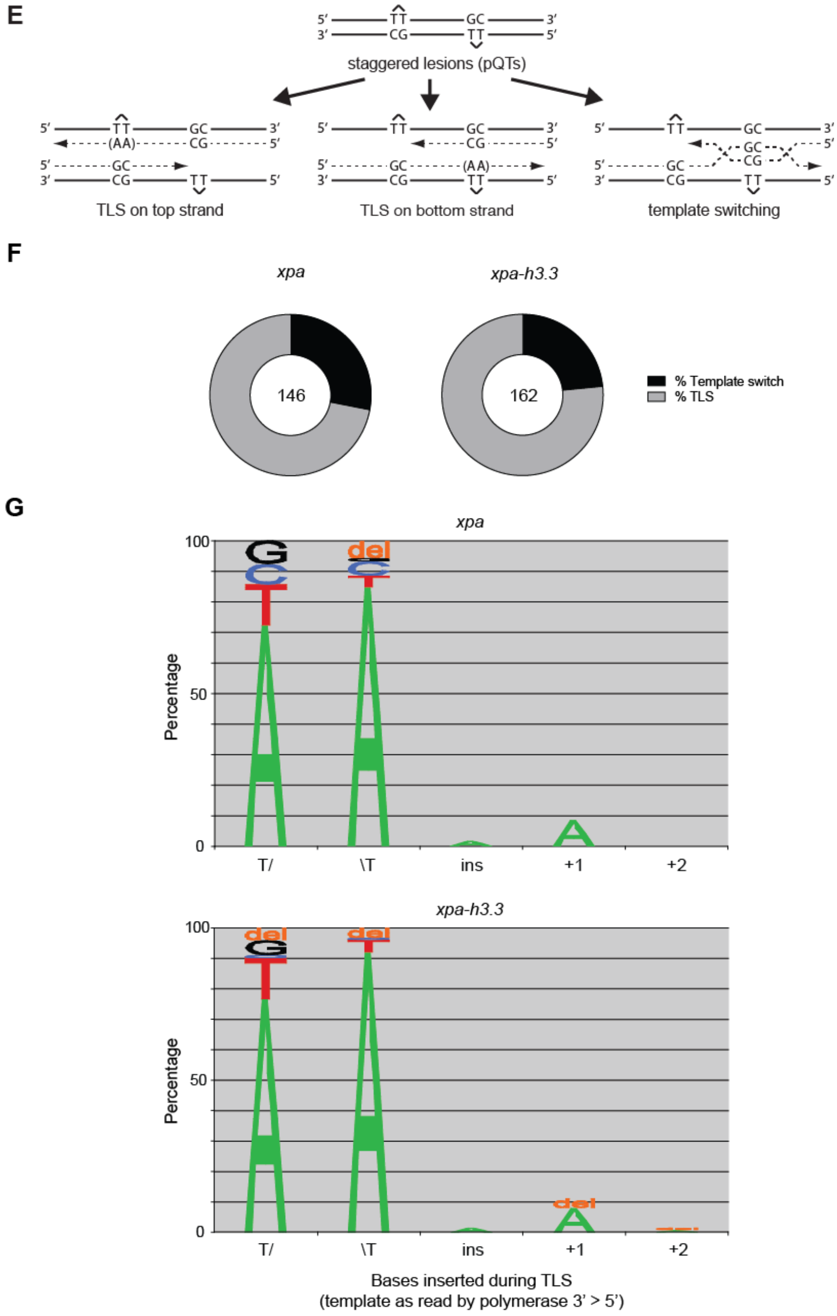

Figure S3 (continued). (Related to Figure 3). Bypass of (6-4)-photoproducts in *h3.3* cells. E. Schematic of the staggered photoproducts in the replicating plasmid pQ1 and the possible outcomes of their replication [taken from S2]. F. Percentage of lesions replicated by presumed 'template switching' vs. translesion synthesis (TLS). The number in the centre of the pie chart indicates the number of analysed sequences. G. Representation of the bases inserted during TLS. The order follows the template as read by the polymerase, 3' T of lesion (T/), 5' T of lesion (/T), non-templated insertion (extra base), mutation at the +1 and +2 positions [see also S2]. *xpa* is used as a control to eliminate any contribution of excision repair, which would give the same outcome as template switching.

Figure S4

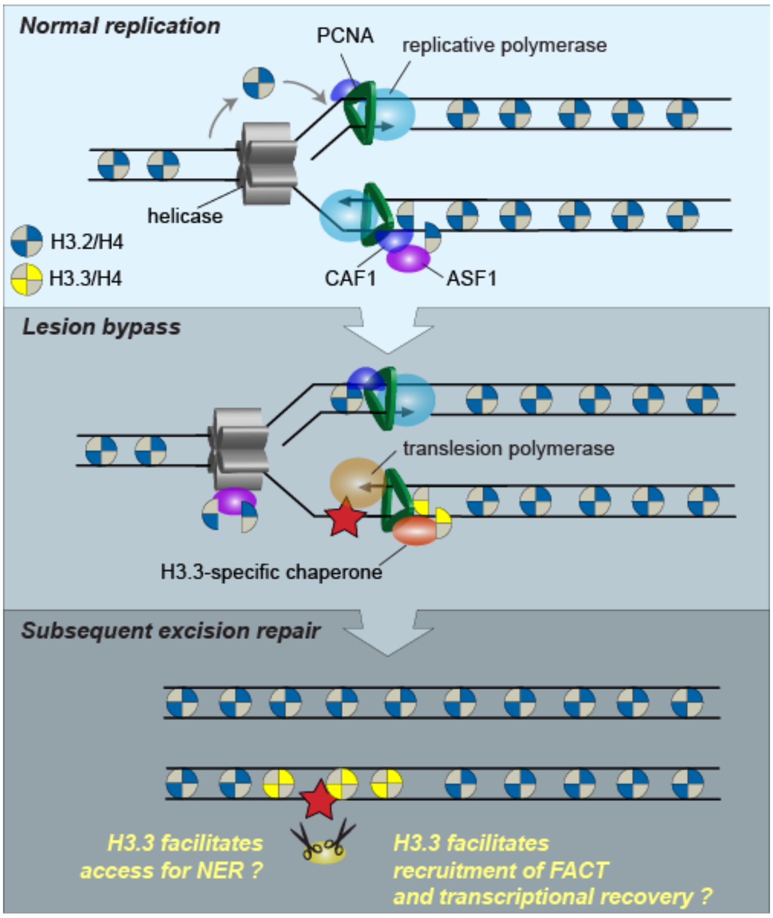

Figure S4. A theoretical model for the role of H3.3 during DNA damage bypass and excision repair. During normal replication both recycled H3/H4 and newly synthesised H3.2/H4 are incorporated into the nascent daughter strands, chaperones by CAF1 and ASF1 (Top panel). When the fork encounters a lesion (Red star, middle panel) we propose that the histone chaperone switches to one of the H3.3-specific chaperones (for instance HIRA or ATRX/DAXX). The absence of H3.3 in these circumstances causes a delay in fork progression. We suggest that, under normal circumstances, H3.3 is incorporated as the fork bypasses the lesion preventing any delay and marking the site of lesion bypass with H3.3. We propose that the presence of H3.3 either improves access for the NER apparatus and / or facilitates transcriptional recovery after repair (Lower panel).

Table S1. List of genes significantly up or down-regulated in *h3.3* vs. wildtype RNA-seq. (Related to Figure 1). Separate Excel spreadsheet listing the genes upregulated and downregulated more than 2-fold with a p value of < 0.001. The complete RNA-seq datasets (three each of wild type and *h3.3*) have been deposited in Array Express (https://www.ebi.ac.uk/arrayexpress/) with accession number E-MTAB-2754.

Supplemental Experimental Procedures

DT40 cell culture & complementation

Wild type DT40 and its derivatives were cultured as previously described [S3]. Growth was monitored with a Vi-Cell counter (Beckman-Coulter). Generation of lines stably expressing H3.3-GFP was performed as described [S4]. Briefly, 2 $x10^7$ cells were transfected with 20 μg plasmid DNA (see 'H3.3 constructs and site directed mutagenesis' below) in a BioRad Gene Pulser with 0.4mm cuvettes at 250 V, 950 μF. Drug resistant clones were expanded and assessed for GFP expression. Clones were selected to have matched GFP expression (Figure S2).

Gene targeting

H3.3B

To delete the H3.3B gene we created a targeting construct by amplifying a genomic region including the entire H3.3B gene with primers H33BF1 and H33BR1. The PCR product was cloned into pBluescript and the EcoRV fragment that contains most of the coding region was replaced with a selection cassette (blasticidin/puromycin) by blunt ligation. Drug-resistant clones were screened for targeted integration by digestion of genomic DNA with NcoI followed by Southern blotting with a probe 5' of the targeting construct generated with the primers H3.3B-Probe-F and H3.3B-Probe-R. Two homozygous $H3.3^{-/-}$ clones were obtained, one of which was taken forward for targeting of the *H3.3A* locus.

H3.3A

To disrupt the H3.3A gene we created a targeting construct by amplifying genomic regions up- and downstream of the H3.3A coding region with primers H33AF1 and H33AR1, H33AF2 and H33AR2. The 5' arm was cloned into TOPO and moved to pBluescript as an ApaI fragment. The PCR product of the 3' arm was digested with SacI and cloned into pBluescript. A selection cassette (conferring blasticidin resistance) was inserted to replace the entire coding region of H3.3A. Drug-resistant clones were screened for targeted integration by PCR with the primers H33A-1-F and H33A-1-R for the first allele, and H33A-2-F and H33A-2-R for the second allele. We obtained two *h3.3* lines, c20 and c32. Both behaved identically. c20 was used for most studies except where stated.

XPA

To delete the XPA gene we used a previously described puromycin resistant targeting construct [S5]. Clones were screened for targeted integration by PCR with the primers XPAF and XPAR.

Colony survival assays

Colony survival experiments were carried out in methylcellulose medium as previously described [S6]. UV light at 254 nm was delivered using a custom-made shuttered source and calibrated with a UV radiometer (UVP, Upland, CA 91786, USA). Cisplatin (CDDP) and methyl methanesulphonate were obtained from Sigma. The D_{10} (dose resulting in a 10% survival) was calculated for each curve and the fold sensitivity of each mutant relative to wild type is given in the relevant figure legend.

H3 constructs and site directed mutagenesis

The pCDH expression vectors which express H3.2 and H3.3 with GFP fused to the C-terminus [S7] were kindly provided by Dr. Simon Elsässer. Site directed mutagenesis of H3.3 was performed as previously described [S8]. Primers used to generate the H3.3[AIG>SVM], H3.3[S31A], H3.3[S31D], H3.3[K27M], H3.3[G34R] and H3.3[G34V] constructs are listed below.

RNA deep sequencing for gene expression analysis

Three wild type and three *h3.3* DT40 pools were expanded for 3 weeks after which RNA was extracted using an RNeasy Mini Kit (Qiagen). The RNA quality and quantity was determined using an Agilent 2100 Bioanalyzer (Agilent Technologies). Sequencing of RNA from each pool was carried out by BGI (Beijing Genomics Institute) using an Illumina HiSeq2000. FASTQ files were aligned to the Chicken cDNA derived from the Galgal4 genome assembly (from:ftp://ftp.ensembl.org/pub/release-74/fasta/gallus_gallus/cdna/) using Bowtie [S9]. A maximum of two mismatches per read were allowed. Only sequences that mapped to one location on the genome were retained. Read counts were identified for each mapped transcript. The DEseq package [S10] from Bioconductor (http://bioconductor.org/) was then used to normalise read counts. Differential expression and associated statistical significance was computed using a negative binomial test from the DESeq package. Graphical representations and further analysis were performed using homemade R scripts. The mapping of dysregulated genes to the chicken karyotype was performed using Ensembl (http://www.ensembl.org/index.html).

Western blotting

Cells were lysed in extraction buffer (50 mM Tris-HCl, pH 8, 250 mM NaCl, 20 mM EGTA, 50 mM NaF, and 1% Triton X-100) and Complete Protease Inhibitor Cocktail (Roche) on ice for 20 minutes. Lysates were cleared by centrifugation at 13’000 rpm for 20 min at 4°C. Extracts were boiled in Laemmli buffer for 5 minutes. Protein

levels were quantified before loading on NuPAGE gels (Life Technologies) and transferred on nitrocellulose membranes (Whatman). Antibodies used at a 1/1000 dilution: anti-Histone H3 (Abcam, ab1791), anti-Histone H3.3 (EMD Millipore, 09-838). Antibodies used at a 1/2000 dilution: anti-mouse (Jackson ImmunoResearch, 115-035-174), anti-rabbit (Jackson ImmunoResearch, 211-032-171).

Flow cytometry

Flow cytometry to assess GFP expression was performed using an LSRII cytometer (BD Biosciences).

Flow-cytometric analysis of cell-cycle progression

In order to monitor the cell cycle after UV treatment (3 J/m^2), cells were incubated 30 minutes before the indicated time with 50μM BrdU. Cells were then placed in 3 volumes of ice cold 1X PBS, spun down at 400g, washed once in cold 1X PBS and then fixed in 75% EtOH for a minimum of 24 hours at -20ºC. Each sample was then spun down and incubated in 15 mM pepsin/30 mM HCl for 20 min at 37°C. The DNA was denatured in 2M HCl for 20 min at room temperature. Cells were then washed once in 1X PBS and resuspended in antibody dilution buffer (0.75% FCS, 0.25% chicken serum, 0.5% Tween 20, 20 mM HEPES in PBS). The pellets were then sequentially incubated for 1h in 1/5 mouse anti-BrdU (Becton Dickinson), 30 minutes 1/50 rabbit anti-mouse Alexa-Fluor 594 (Life Technologies), and 30 minutes 1/50 donkey anti-rabbit Alexa-Fluor 594 (Life Technologies). Total DNA was stained in 1μM Hoechst 33342. Analyses were carried out on a LSRII (BD Sciences). 50,000 unique cells were counted for each sample.

Preparation, spreading and immunolabelling of DNA Fibres

This method is essentially that used in [S11], but with modifications. Exponentially growing DT40 cells (6 x10^6) were incubated at 37 °C with 50 μM IdU for 20 min. They were then spun down and incubated at 37 °C with 50 μM CldU for 20 min. For UV treatment, after labelling with IdU cells were irradiated with 40 J/m^2 254 nm light in 1 ml of PBS and then incubated with CldU as above. Labelled cells were resuspended in PBS to a concentration of 1 × 10^6 cells ml^{-1}. Three μl were spotted onto clean glass Superfrost slides and lysed with 7 μl of 0.5% SDS in 200 mM Tris-HCl (pH 5.5) and 50 mM EDTA (5 min, 20°C). Slides were tilted at 15° to horizontal, allowing the DNA to run slowly down the slide. Slides were then air dried and fixed in 3:1 methanol/acetic acid, and stored at 4°C before immunolabelling.

The DNA fibre spreads were hydrated with water and then denatured with 2.5M HCl for 1hr at 20°C. Slides were washed three times in PBS, then incubated in PBS containing 1% BSA and 0.1% Tween 20 for 1 hr at 20°C. Slides were incubated (45 min, 20 °C) with rat anti-BrdU (Oxford Biotechnology Ltd.) at 1:500 to detect CldU. Slides were then washed three times in PBS and incubated (20 min, 20 °C) with AF488 chicken anti-rat antibody (Invitrogen, Life Technologies) at 1:100. Slides were washed three times in PBS and incubated (20 min, 20°C) with AF488 goat anti-

chicken antibody (Invitrogen, Life Technologies) at 1:100. The slides were then again washed three times with PBS and incubated (45 min, 20°C) with mouse anti-BrdU (BD Biosciences) at 1:10 to detect IdU. Slides were washed three times in PBS and incubated (20 min, 20°C) with AF594 rat anti-mouse antibody (Invitrogen, Life Technologies) at 1:100. Slides were washed three times in PBS and incubated (20 min, 20°C) with AF594 donkey anti-rat antibody (Invitrogen, Life Technologies) at 1:100. Finally, slides were washed three times in PBS and mounted in Fluoromount G (Southern Biotechnology). Slides were kept at 4°C and imaged using a Nikon C1-si confocal microscope. Tract lengths were measured using Adobe Photoshop.

For analysis of DNA replication dynamics (fork velocity and origin density) we additionally revealed the DNA with an anti-DNA antibody as previously described [S1].

Replicating plasmid assay

The replicating plasmid assay was carried out as previously described [2]. The 6-4 photoproduct in a staggered configuration was used in pQ1. The lesion containing oligos were provided by Professor Shigenori Iwai, Osaka University.

Oligonucleotides

Name	Sequence (5' to 3')
H33AF1	CCCTCTGTTGGATGTAGGACA
H33AR1	CCGTGGACTTCATTTAGAGCA
H33AF2	TGGGTAGAGTCTGGAGCTGAA
H33AR2	CCTCTTGGTGTGAAGCAGAAC
H33A-1-F	TGGTTTGTCCAAACTCATCAA
H33A-1-R	CACAGTGCCATTTGGGTTTA
H33A-2-F	AAGGGCCTTCTCTCTGTTAGC
H33A-2-R	CACAGTGCCATTTGGGTTTA
H33BF1	ACCTCAGGGCAGGTGACACAAAACC
H33BR1	GGTGTTCTACTGATGGAAAGGGGAG
H3.3B-Probe-F	CTACTGATGGAAAGGGGAGATAGG
H3.3B-Probe-R	TAAGCCTAAGCTGGTGTCCTGAGAG
XPAF	GGTGGGGCTGATAGTGTGTAA
XPAR	GATGGAGGAACGAACTGACAA
K27MF	GCCGCCCGCATGAGCGCCCCG
K27MR	CGGGGCGCTCATGCGGGCGGC
S31AF	GAGCGCCCCGGCCACCGGCGG
S31AR	CCGCCGGTGGCCGGGGCGCTC
S31DF	CAAGAGCGCCCCGGACACCGGCGGCGTG
S31DR	CACGCCGCCGGTGTCCGGGGCGCTCTTG
G34RF	CCGTCCACCGGCAGGGTGAAGAAGCCTC
G34RR	GAGGCTTCTTCACCCTGCCGGTGGACGG

G34VF	CCGTCCACCGGCGTGGTGAAGAAGCCTC
G34VR	GAGGCTTCTTCACCACGCCGGTGGACGG
AIGF	CTCCTGCAGAGCCATGACGGCCGAGCTCTGGAAGCGC
AIGR	GCGCTTCCAGAGCTCGGCCGTCATGGCTCTGCAGGAG

Supplemental References

S1. Guilbaud, G., Rappailles, A., Baker, A., Chen, C.-L., Arneodo, A., Goldar, A., d'Aubenton-Carafa, Y., Thermes, C., Audit, B., and Hyrien, O. (2011). Evidence for sequential and increasing activation of replication origins along replication timing gradients in the human genome. PLoS Comput. Biol. *7*, e1002322.

S2. Szüts, D., Marcus, A. P., Himoto, M., Iwai, S., and Sale, J. E. (2008). REV1 restrains DNA polymerase zeta to ensure frame fidelity during translesion synthesis of UV photoproducts in vivo. Nucleic Acids Res. *36*, 6767–6780.

S3. Simpson, L. J., and Sale, J. E. (2003). Rev1 is essential for DNA damage tolerance and non-templated immunoglobulin gene mutation in a vertebrate cell line. EMBO J. *22*, 1654–1664.

S4. Sale, J. E. (2006). Stable non-targeted transfection of DT40. Subcell. Biochem. *40*, 341–344.

S5. Okada, T., Sonoda, E., Yamashita, Y. M., Koyoshi, S., Tateishi, S., Yamaizumi, M., Takata, M., Ogawa, O., and Takeda, S. (2002). Involvement of vertebrate polkappa in Rad18-independent postreplication repair of UV damage. J. Biol. Chem. *277*, 48690–48695.

S6. Simpson, L. J., and Sale, J. E. (2006). Colony survival assay. Subcell. Biochem. *40*, 387–391.

S7. Elsässer, S. J., Huang, H., Lewis, P. W., Chin, J. W., Allis, C. D., and Patel, D. J. (2012). DAXX envelops a histone H3.3-H4 dimer for H3.3-specific recognition. Nature *491*, 560–565.

S8. Ross, A.-L., Simpson, L. J., and Sale, J. E. (2005). Vertebrate DNA damage tolerance requires the C-terminus but not BRCT or transferase domains of REV1. Nucleic Acids Res. *33*, 1280–1289.

S9. Langmead, B., Trapnell, C., Pop, M., and Salzberg, S. L. (2009). Ultrafast and memory-efficient alignment of short DNA sequences to the human genome. Genome Biol. *10*, R25.

S10. Anders, S., and Huber, W. (2010). Differential expression analysis for sequence count data. Genome Biol. *11*, R106.

S11. Edmunds, C. E., Simpson, L. J., and Sale, J. E. (2008). PCNA ubiquitination and REV1 define temporally distinct mechanisms for controlling translesion synthesis in the avian cell line DT40. Molecular Cell *30*, 519–529.
