# Supplementary material for: Histone H3.3 Is Required to Maintain Replication Fork Progression after UV Damage
Source: Curr Biol. 2014 Sep 22;24(18):2195–201. doi: 10.1016/j.cub.2014.07.077 (PMC4175177; doi:10.1016/j.cub.2014.07.077)
Supplement: Document S2. Article plus Supplemental Information [file mmc3.pdf]

Current Biology 24, 2195–2201, September 22, 2014 ©2014 The Authors http://dx.doi.org/10.1016/j.cub.2014.07.077

Report

Histone H3.3 Is Required to Maintain Replication Fork Progression after UV Damage

Alexander Frey,[1] Tamar Listovsky,[1] Guillaume Guilbaud,[1] Peter Sarkies,[1,2] and Julian E. Sale[1,*]
[1]Medical Research Council Laboratory of Molecular Biology, Francis Crick Avenue, Cambridge CB2 0QH, UK

Summary

Unlike histone H3, which is present only in S phase, the variant histone H3.3 is expressed throughout the cell cycle [1] and is incorporated into chromatin independent of replication [2]. Recently, H3.3 has been implicated in the cellular response to ultraviolet (UV) light [3]. Here, we show that chicken DT40 cells completely lacking H3.3 are hypersensitive to UV light, a defect that epistasis analysis suggests may result from less-effective nucleotide excision repair. Unexpectedly, H3.3-deficient cells also exhibit a substantial defect in maintaining replication fork progression on UV-damaged DNA, which is independent of nucleotide excision repair, demonstrating a clear requirement for H3.3 during S phase. Both the UV hypersensitivity and replication fork slowing are reversed by expression of H3.3 and require the specific residues in the α2 helix that are responsible for H3.3 binding its dedicated chaperones. However, expression of an H3.3 mutant in which serine 31 is replaced with alanine, the equivalent residue in H3.2, restores normal fork progression but not UV resistance, suggesting that H3.3[S31A] may be incorporated at UV-damaged forks but is unable to help cells tolerate UV lesions. Similar behavior was observed with expression of H3.3 carrying mutations at K27 and G34, which have been reported in pediatric brain cancers. We speculate that incorporation of H3.3 during replication may mark sites of lesion bypass and, possibly through an as-yet-unidentified function of the N-terminal tail, facilitate subsequent processing of the damage.

Results and Discussion

H3.3-Deficient DT40 Cells Are Viable but Exhibit Alterations in Gene Expression

H3.3 is incorporated throughout the cell cycle [2, 4], particularly in regions of the genome in which histones need to be displaced, such as transcribed genes or regulatory elements [5, 6]. Incorporation in these contexts depends on the histone chaperone HIRA [7] and helps maintain chromatin structure by filling gaps left by loss of H3.1/H4 [5, 8]. H3.3 deposition at transcriptionally active loci has also been proposed to help maintain active expression, possibly by creating a more accessible chromatin structure [2, 9]. However, H3.3 is also incorporated in some repressed loci and at telomeres and pericentric heterochromatin, where deposition depends on the ATRX-DAXX chaperone complex [10–12]. Although H3.3 is not essential for transcription in *Drosophila*, its loss results in significantly decreased fertility and reduced viability during embryogenesis [13]. Mouse embryonic stem cells with no H3.3B and depleted of H3.3A exhibit altered regulation of polycomb-dependent gene expression that interferes with their ability to differentiate [14]. Mice lacking H3.3B exhibit a semi-lethal phenotype with reduced growth, anaphase bridging, and karyotypic abnormalities [15]. Recently, H3.3 has also been implicated in the response to ultraviolet (UV) irradiation, because its chaperone HIRA is required to promote transcription restart after UV damage [3].

In order to examine the effect of complete loss of H3.3 in a differentiated vertebrate cell line, we created an H3.3 null variant of the chicken bursal lymphoma DT40 [16]. In chicken, as in mammals, H3.3 is encoded by two loci, *H3.3A* on chromosome 18 and *H3.3B* on chromosome 3. Despite considerable divergence of the cDNA sequence of H3.3A and H3.3B, they encode identical proteins, which also have the same sequence as human H3.3. RNA deep-sequencing analysis (RNA-seq) of DT40 revealed that H3.3B contributes over 90% of the total pool of H3.3 transcript in chicken DT40 B cells (Figure 1A).

To create H3.3 null DT40 cells, we first disrupted both alleles of *H3.3B* by homologous recombination using a targeting strategy that removed the majority of the coding sequence (Figure 1B; Supplemental Experimental Procedures available online). This resulted in a substantial reduction of total H3.3 protein levels (Figure 1C), as predicted by the RNA-seq data (Figure 1A). We then disrupted both alleles of *H3.3A* by removing the whole H3.3A coding sequence (Figure 1B). This resulted in loss of the remaining H3.3 protein (Figure 1C). We subsequently refer to this H3.3 null line as *h3.3* DT40. *h3.3* cells proliferate more slowly than wild-type (c. 15 versus c. 11 hr; Figure 1D). Their unperturbed cell-cycle profile suggests that this is at least in part explained by an increase in spontaneous apoptosis (Figure 1E).

We next examined the extent of transcriptional dysregulation in cells lacking H3.3 by RNA-seq. This analysis revealed that 557 of 16,396 gene transcripts (3.4%) exhibited a >2-fold and significant ($p < 0.001$) change in expression (Figure 1F; Table S1). Interestingly, the number of genes exhibiting a significant decrease in expression (235) is actually slightly exceeded by those increasing in expression (324), supporting recent evidence that H3.3, or its modifications, is not just important for actively expressed loci [12, 14]. We observed no underlying pattern to the chromosomal locations of affected genes (Figure S1). Thus, loss of H3.3 is linked to significant changes in gene expression, but affects a relatively small fraction of loci in DT40 cells.

H3.3 Is Likely to Operate in Concert with the Nucleotide Excision Repair Pathway

In addition to being incorporated during transcription, recent experiments have shown that H3.3 is deposited at sites of UV-induced DNA damage by the histone chaperone HIRA, where it facilitates the recovery of transcription after repair of the damage [3]. We therefore asked whether *h3.3* cells exhibit sensitivity to UV light. *h3.3* cells were modestly, but

[2]Present address: The Gurdon Institute, Tennis Court Road, Cambridge CB2 1QR, UK
*Correspondence: jes@mrc-lmb.cam.ac.uk
This is an open access article under the CC BY license (http://creativecommons.org/licenses/by/3.0/).
CrossMark

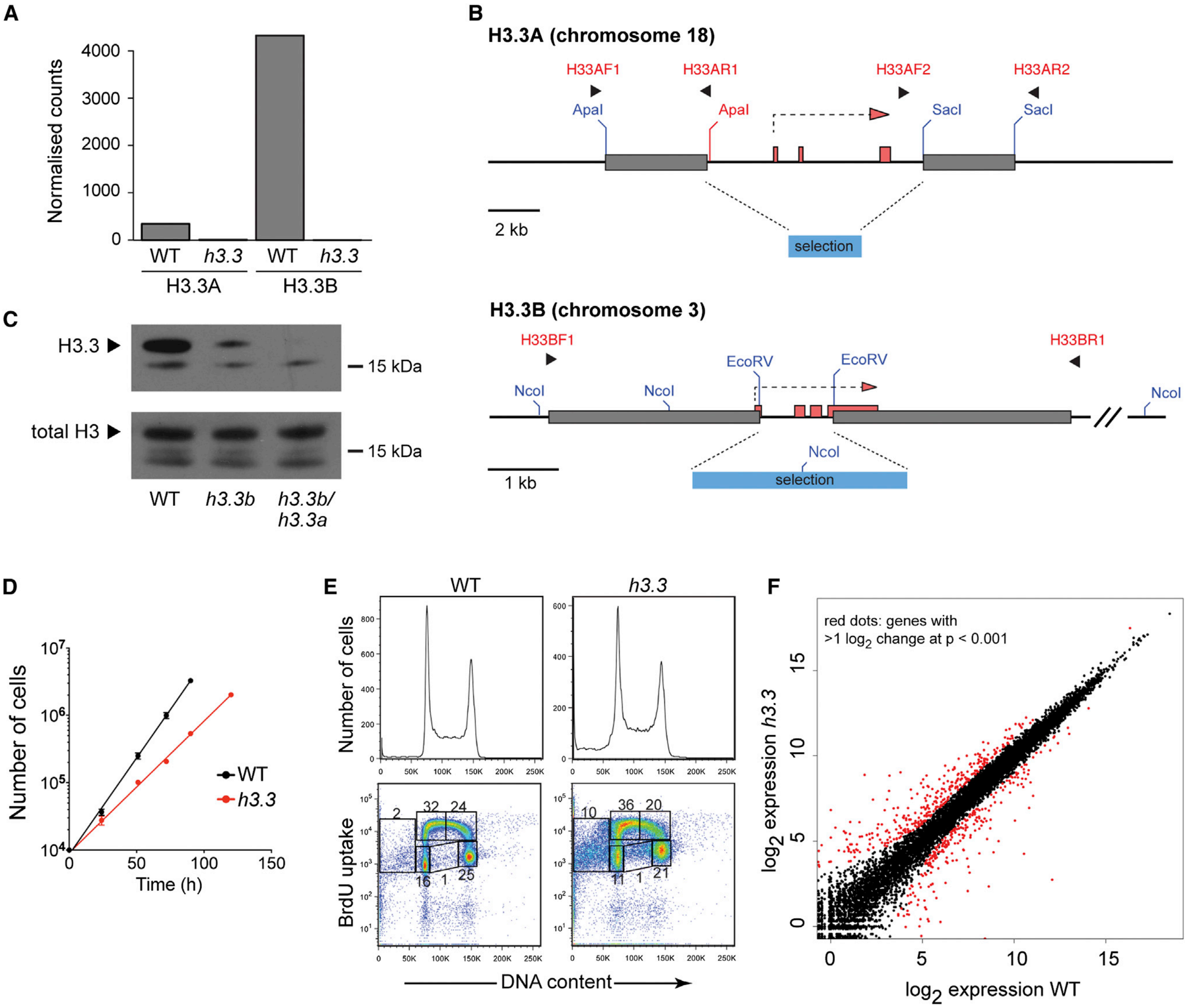

Figure 1. DT40 Cells Deficient in H3.3

(A) Expression of H3.3 from the two alleles H3.3A and H3.3B, monitored by RNA-seq. The y axis represents the normalized number of reads from each locus.
(B) Gene targeting strategies for the *H3.3A* and *H3.3B* loci. Exons are shown as salmon pink boxes. The targeting arms are shown as gray boxes and the selection cassette as a blue box. Primers are indicated in red and key restriction sites are in blue (endogenous) or red (introduced during cloning). See also Supplemental Experimental Procedures.
(C) Confirmation of loss of H3.3 expression. Western blot of acid-extracted histones for H3.3 and total H3 from wild-type, cells lacking H3.3B (*h3.3b*), and cells lacking both H3.3A and H3.3B (*h3.3b*/*h3.3a*; abbreviated *h3.3*).
(D) Growth of wild-type and *h3.3* cells. Each point represents the average cell number for three experiments with error bars showing 1 SD. The lines are a linear regression fit.
(E) One- and two-dimensional cell-cycle analysis of asynchronous populations of wild-type and *h3.3* DT40. Each plot shows a total of 50,000 cells, and the percentage of the total cycling cells in each gate is indicated. BrdU, bromodeoxyuridine.
(F) RNA-seq from wild-type versus *h3.3*. The $\log_2$ expression level for each gene is determined from the normalized counts of three wild-type and three H3.3 RNA-seq experiments. Red dots represent genes whose expression differs by greater than 2-fold with $p < 0.001$.
See also Figure S1 and Table S1.

consistently, hypersensitive to UV irradiation (Figure 2A). This is unlikely to be a secondary effect, because no known DNA damage response genes exhibited significantly dysregulated expression in *h3.3* cells (Table S1). Further, the sensitivity of *h3.3* cells to UV light was reversed by stable expression of H3.3 C-terminally tagged with GFP (Figure 2A; Figure S2). H3.2 could not substitute for H3.3 in rescuing the UV sensitivity of *h3.3*. In fact, ectopic expression of H3.2 appeared to cause further sensitization to UV, as previously observed in yeast [17]. Because H3.3 has been implicated in processes related to nucleotide excision repair (NER) [3], we examined its genetic relationship to NER by performing epistasis analysis of H3.3 with XPA, a key component of the NER pathway. *xpa* DT40 cells are highly sensitive to UV light, considerably more so than *h3.3* (Figure 2B). A double *h3.3*/*xpa* mutant was no more sensitive than *xpa* alone, suggesting that XPA may be epistatic to H3.3 and that H3.3 acts to facilitate excision repair of a subset of UV lesions.

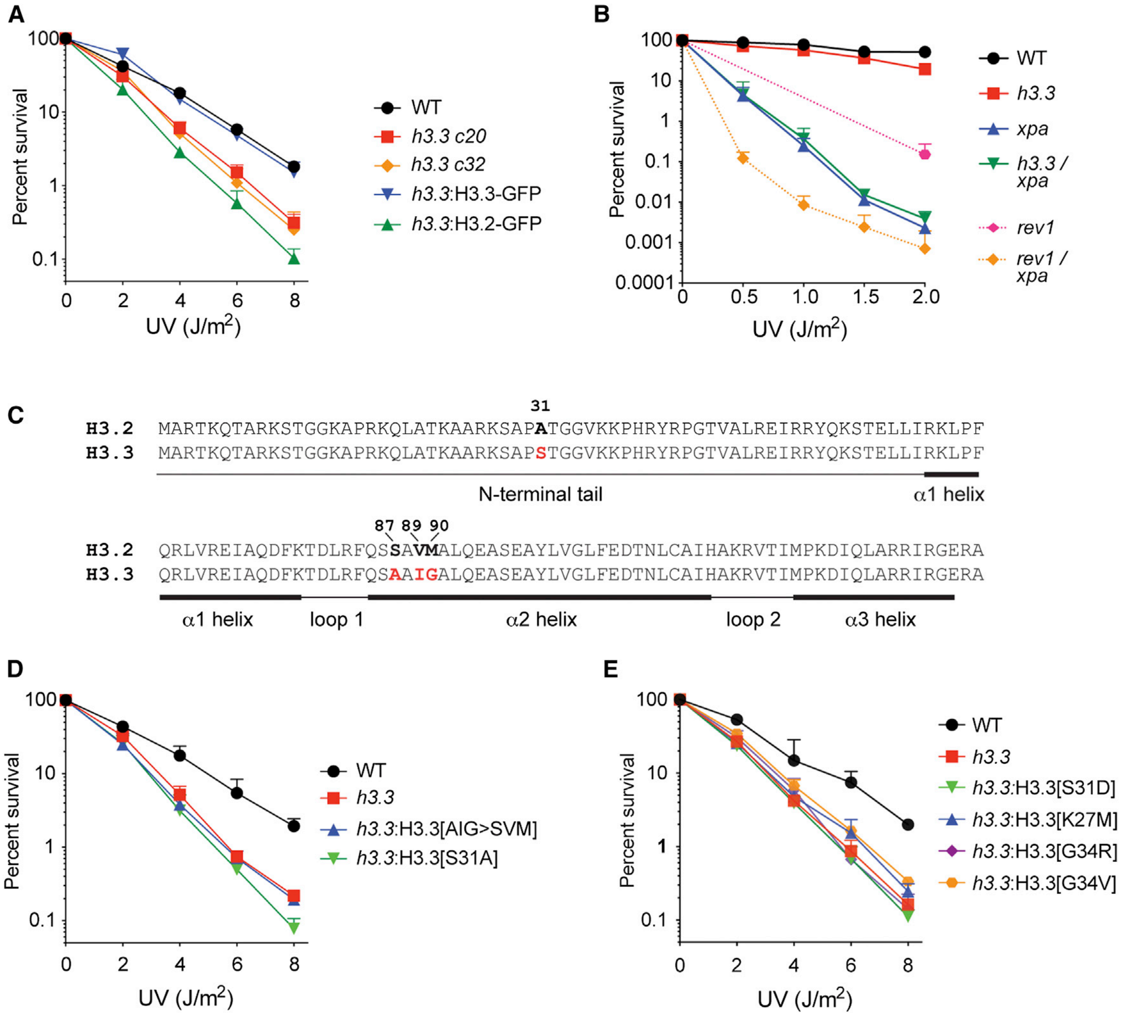

Figure 2. DNA Damage Sensitivity of H3.3-Deficient Cells

Colony survival assays following exposure to UV light.

(A) Complementation of the DNA damage sensitivity of *h3.3* cells with H3.3 and H3.2. Two clones of the *h3.3* knockout (c20 and c32) are shown. Fold sensitivities versus wild-type: *h3.3 c20* 1.4; *h3.3 c32* 1.4; *h3.3*:H3.3-GFP 1; *h3.3*:H3.2-GFP 1.7.

(B) Epistasis of XPA to H3.3. That the colony survival assay has the power to detect additional sensitivity beyond that of the *xpa* mutant is demonstrated by a *rev1/xpa* mutant, which lacks both NER and tolerance of UV lesions during replication by translesion synthesis. Fold sensitivities versus wild-type: *h3.3* 2.1; *xpa* 17.5; *h3.3/xpa* 16.7; *rev1* 9; *rev1/xpa* 310.

(C) Alignment of chicken H3.2 and H3.3. The key differences are highlighted and the domain structure of the protein is indicated below the alignment.

(D) Complementation of *h3.3* cells with H3.3 with a mutated chaperone-binding patch (abbreviated AIG>SVM) or S31A. Fold sensitivities versus wild-type: *h3.3* 1.6; *h3.3*:H3.3[AIG>SVM] 1.7; *h3.3*:H3.3[S31A] 1.8.

(E) Effect of expression of H3.3 carrying a potentially phosphomimetic mutation of S31, S31D, or three nearby cancer-associated mutations, H3.3[K27M], H3.3[G34R], and H3.3[G34V]. Fold sensitivities versus wild-type: *h3.3* 1.7; *h3.3*:H3.3[S31D] 1.8; *h3.3*:H3.3[K27M] 1.6; *h3.3*:H3.3[G34R] 1.6; *h3.3*:H3.3[G34V] 1.5.

Survival assays were performed three times and 1 SD of the surviving fraction is indicated. For clarity, only the positive error bar is shown. See also Figure S2.

However, although the UV colony survival assay has the dynamic range to detect additional sensitivity over and above that of the *xpa* mutant (Figure 2B), the very large difference in the sensitivities of the *h3.3* and *xpa* mutants means that epistasis in this instance must be interpreted with some caution.

Resistance to DNA Damage Requires the H3.3-Specific Chaperone-Binding Patch and S31

H3.3 differs at two sites from H3.2, the single canonical H3 in chickens (Figure 2C). S31, in the N-terminal tail region and an alanine in H3.2, has been reported to be phosphorylated during mitosis, although the function of this modification is not yet understood [18]. H3.3 also has three residues at the base of α helix 2 that differ from H3.2. These are A87/I89/G90, which are S87/V89/M90 in H3.2 (hereafter referred to as “AIG” and “SVM”). This “patch” is thought to define the chaperone specificity of H3.2 and H3.3. Thus, the AIG patch is required for binding of H3.3 to DAXX [10] and is required for replication-independent chromatin deposition [2, 4], that is dependent on HIRA [7]. We created *h3.3* clones stably expressing H3.3-GFP carrying either a substitution of the AIG patch with the

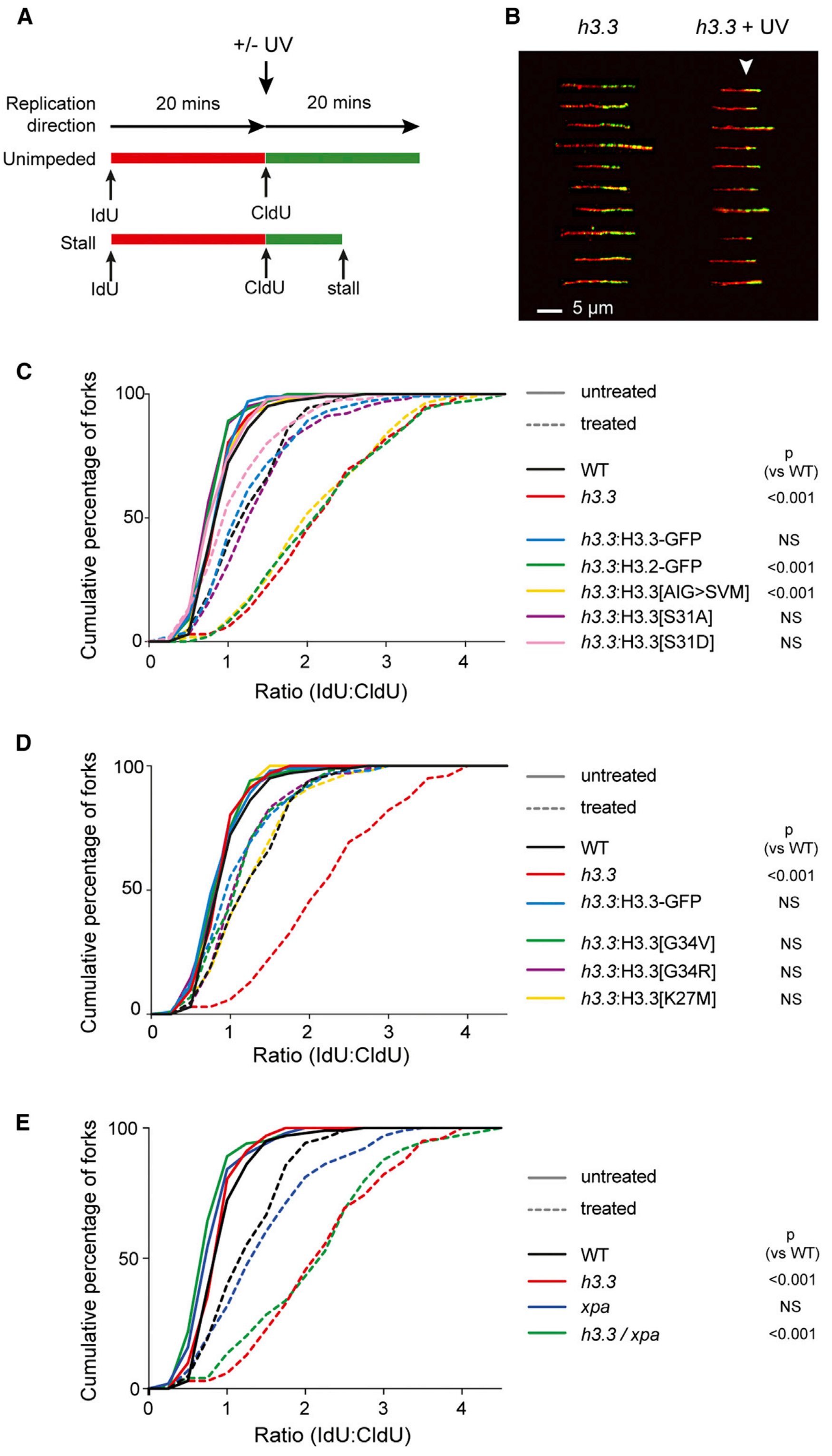

Figure 3. H3.3 Is Required to Maintain Replication Fork Progression after UV Exposure

(A) Schematic of the fork labeling experiment. IdU, iododeoxyuridine; CldU, chlorodeoxyuridine.

(B) Sample pictures of DNA fibers labeled from *h3.3* cells. The point of UV exposure is indicated by the white arrowhead.

(C–E) Replication fork stalling of wild-type or mutants in response to either sham irradiation (solid lines) or 40 J/m^2 265 nm UV light (dashed lines). The ratio of the length of the second label to the first is calculated for each fiber, and the data are presented as a cumulative percentage of forks at each IdU:CldU ratio. The p value that the cumulative distribution with UV is different from wild-type is shown (Kolmogorov-Smirnov test). NS, not significant (i.e., p > 0.001).

See also Figure S3.

SVM patch of H3.3 or an S31A substitution and ensured matched expression levels by monitoring GFP by flow cytometry (Figure S2). Neither the AIG patch nor S31A H3.3 mutants complemented the UV hypersensitivity of *h3.3* cells (Figure 2D), suggesting that the chaperone binding specificity of H3.3 and a serine at position 31 are required. A potentially phosphomimetic substitution of S31 with aspartic acid also did not complement the UV sensitivity of *h3.3* cells (H3.3[S31D]; Figure 2E).

Pediatric Cancer-Associated H3.3 Mutations near S31 Also Result in UV Sensitivity

Recently, mutations in the N-terminal tail of H3.3, in the vicinity of S31, have been linked to a number of pediatric cancers, including glioblastoma, chondroblastoma, and giant cell tumors of bone [19–21]. Understanding the mechanistic basis for the clinical effects of these apparently driver mutations has focused on their effects on posttranslational modifications of H3. Thus, mutations at G34 affect the global distribution of H3K36me3 and changes in gene expression [22]. Likewise, mutation of H3.3K27, a residue whose trimethylation is associated with polycomb complex-mediated transcriptional repression, results in reduced global H3K27me3 and derepression of multiple transcripts [23]. Because S31 lies close to these residues, we wondered whether the cancer-associated mutations K27M, G34R, and G34V [19, 20] might also confer sensitivity to DNA damage. Interestingly, all three H3.3 mutants exhibit UV sensitivity similar to the *h3.3* knockout, suggesting that these residues are also required for the role played by H3.3 in facilitating excision repair (Figure 2E). This somewhat surprising result suggests the possibility that H3.3 cancer-associated mutations could impact on DNA repair as well as on transcriptional regulation, a point that merits further exploration.

H3.3 Is Required during S Phase to Maintain Processive Replication after UV Irradiation

Histone supply affects the processivity of DNA replication [24]. Although the deposition of H3.3 is primarily replication

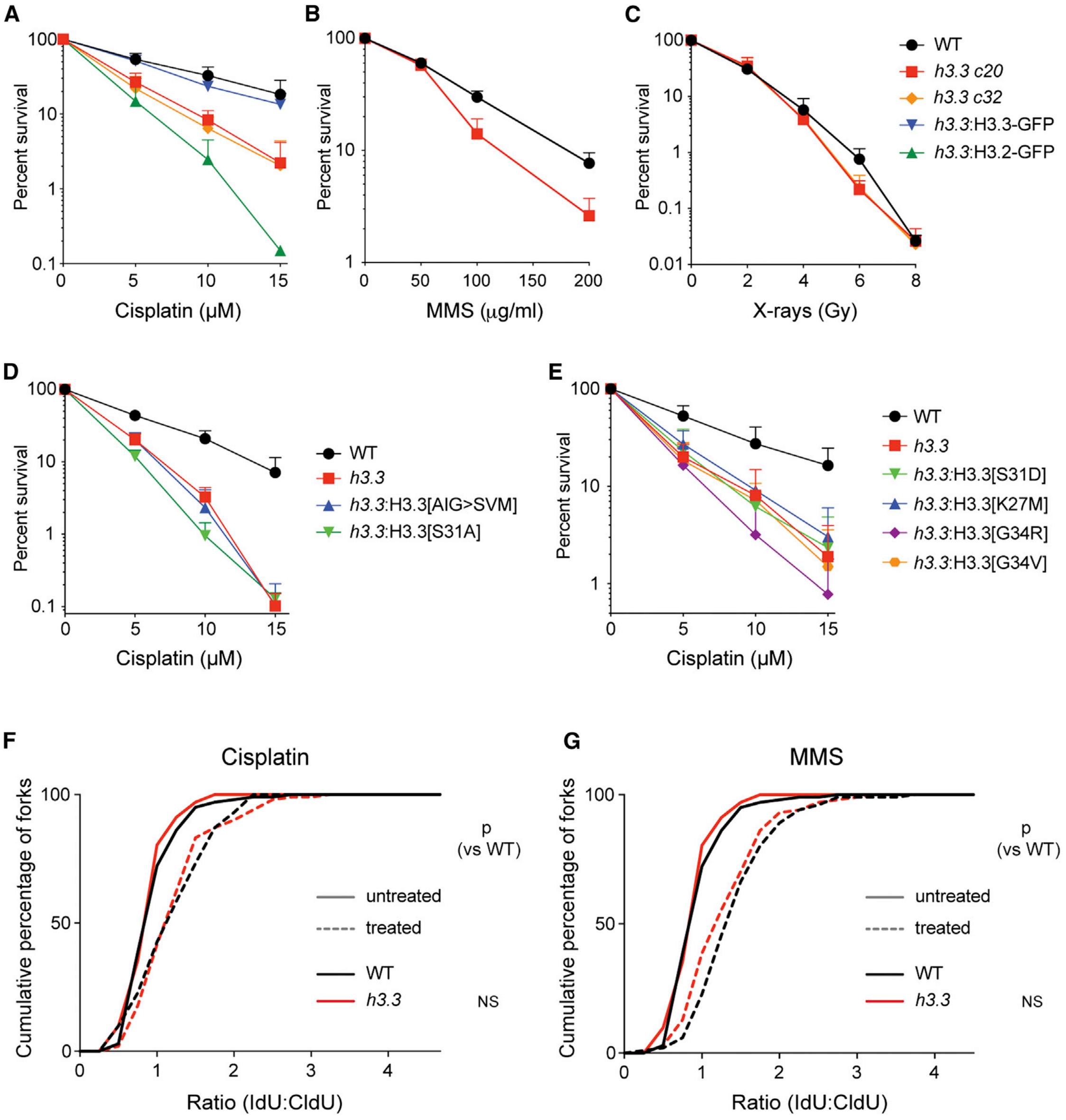

Figure 4. Response of *h3.3* Cells to Other Forms of DNA Damage

(A) Sensitivity of *h3.3* cells to cisplatin. Fold sensitivities versus wild-type: *h3.3 c20* 2.5; *h3.3 c32* 2.8; *h3.3*:H3.3-GFP 1.4; *h3.3*:H3.2-GFP 4.1.

(B) Sensitivity of *h3.3* cells to methyl methanesulfonate. Fold sensitivity versus wild-type: *h3.3* 1.4.

(C) Sensitivity of *h3.3* cells to X-rays. Fold sensitivities versus wild-type: *h3.3 c20* 1.1; *h3.3 c32* 1.1.

(D) Sensitivity of *h3.3* AIG patch and S31A mutants to cisplatin. Fold sensitivities versus wild-type: *h3.3* 2.3; *h3.3*:H3.3[AIG>SVM] 2.3; *h3.3*:H3.3[S31A] 2.6.

(E) Sensitivity of *h3.3* N-terminal tail mutants to cisplatin. Fold sensitivities versus wild-type: *h3.3* 2.2; *h3.3*:H3.3[S31D] 2.1; *h3.3*:H3.3[K27M] 1.9; *h3.3*:H3.3[G34R] 2.7; *h3.3*:H3.3[G34V] 2.3. Survival assays were performed three times and 1 SD of the surviving fraction is indicated. For clarity, only the positive error bar is shown.

(F) Replication fork stalling of wild-type or mutants in response to either sham treatment (solid lines) or 2.5 mM cisplatin (dashed lines).

(G) Replication fork stalling of wild-type or mutants in response to either sham treatment (solid lines) or 0.05% methyl methanesulfonate (dashed lines). The p value that the cumulative distribution with UV is different from wild-type is shown (Kolmogorov-Smirnov test). NS, not significant (i.e., $p > 0.001$).

independent, we asked whether the absence of H3.3 affected replication by monitoring fork progression in stretched DNA fibers. We pulse labeled cells sequentially with two different halogenated nucleosides (iododeoxyuridine and chlorodeoxyuridine; 20 min each), stretched the extracted DNA on glass slides, and revealed the replicons with antibodies specific for the halogenated nucleotides (Figure 3A). Loss of H3.3 did not affect replication dynamics in unperturbed conditions. We observed a small, but not significant, decrease in median fork velocity in *h3.3* cells but no change in replication origin density (Figures S3A–S3C). However, after UV irradiation, applied at the same time as the second label, replication fork progression in the second 20 min was dramatically reduced in *h3.3* cells in comparison to wild-type (Figures 3B and 3C). It is likely that at least some of these forks remain persistently blocked, because a greater fraction of *h3.3* cells

accumulate in late S phase 24 hr after UV exposure, suggesting a delay in completion of replication (Figure S3D). The delayed fork progression following UV exposure in *h3.3* cells was reduced to wild-type levels by expression of H3.3-GFP but not H3.2-GFP (Figure 3C). Although this defect is reminiscent of cells lacking the translesion polymerase REV1 [25, 26], we could observe robust translesion synthesis of UV (6-4) photoproducts in *xpa/h3.3* cells using a replicating plasmid assay [27] and, further, the frame infidelity characteristic of photoproduct bypass in REV1-deficient cells [27] was not evident (Figures S3E–S3G). Thus, delayed replication fork progression after UV damage in *h3.3* cells does not appear to result from a significant defect in REV1-dependent translesion DNA synthesis. We then asked whether the role of H3.3 at the replication fork was also dependent on both the AIG patch and S31, as for UV sensitivity. Whereas the AIG-to-SVM patch mutant failed to complement the defective fork progression after UV (Figure 3C), the H3.3[S31A] mutant restored wild-type behavior (Figure 3C), as did the cancer-associated mutants G34V, G34R, and K27M (Figure 3D). In view of the apparent epistasis of H3.3 and XPA, we considered whether the delayed fork progression in *h3.3* cells reflected defective excision repair. However, *h3.3* cells exhibit a much more prominent defect in fork progression after UV than *xpa* cells, the response of which is similar to wild-type (Figure 3E). This is not consistent with the fork progression defect seen after UV in *h3.3* cells resulting from defective NER at the fork, an event that in any case would likely be deleterious to cell survival due to strand incision at the lesion causing replication fork collapse.

Finally, we asked whether the role of H3.3 in the response to UV was also seen with other forms of DNA damage. In addition to UV, *h3.3* cells exhibit mild hypersensitivity to the interstrand crosslinking agent cisplatin and the alkylating agent methyl methanesulfonate (MMS) but not to X-rays (Figures 4A–4C). In the case of cisplatin, both the AIG patch and N-terminal tail mutants discussed above exhibit hypersensitivity (Figures 4D and 4E), as observed with UV. However, for neither cisplatin nor MMS is there any exacerbation of the delay in fork progression induced by these agents (Figures 4F and 4G), a point we consider further below.

Our observations provide the first clear evidence of the involvement of a variant histone in replication fork progression, and suggest that forks require a supply of H3.3 when they encounter UV damage to maintain processive replication. Although our experiments are not able to show directly that H3.3 is incorporated by the replication fork during replication of UV DNA damage, by analogy with the effect of histone supply on bulk DNA replication [24], we suggest that the defective fork progression in *h3.3* cells is a result of failure of a process that would normally see H3.3 incorporated. We speculate that H3.3 incorporation during the replication of UV lesions at the fork, and possibly during postreplicative lesion bypass, may facilitate subsequent access and repair (Figure S4). H3.3 incorporation would imply the need for an H3.3-specific chaperone. HIRA would seem to be a strong candidate given its documented role in H3.3 incorporation at sites of UV damage [3], although the same study reported that HeLa cells depleted for HIRA are not UV sensitive [3]. Whether ATRX plays any role in replicating UV-damaged DNA is unknown, but it has been implicated in the replication of G quadruplex DNA [28] and, recently, ATRX-deficient cells have been shown to exhibit replication defects, suggesting that it contributes to limiting fork stalling during S phase [29].

Although cells lacking H3.3 are sensitive to UV, MMS, and cisplatin, a fork progression defect, as assessed in labeled DNA fibers, is only observed after UV exposure. This suggests a broad requirement for H3.3 in facilitating DNA repair, but that incorporation of this histone variant may not only take place "on the fly" at the replication fork when it encounters DNA damage but also, for instance, during lesion bypass in postreplicative gaps. Loss of this latter role would not be detectable as a defect in the DNA fiber assay. The basis for this specificity remains unclear, but we speculate that it may be related to the mechanisms and complexes cells bring into play at different lesions, which in turn may affect the timing of lesion bypass [30]. Indeed, such damage-dependent specificity is now well documented in translesion synthesis [31] and, recently, damage caused by UV and by MMS has been shown to induce quite distinct bypass responses in human cells [32]. However, much further work is needed to understand the contexts in which H3.3 is required for processive replication of damaged DNA.

Finally, how might H3.3 incorporation facilitate subsequent DNA repair and survival? Because it has been proposed that H3.3-containing chromatin has a more open and accessible structure [9], its incorporation may be particularly important for promoting NER in highly condensed regions of the genome. Additionally, the damage sensitivity of the *h3.3* cells may also be related to its ability to promote transcriptional recovery after repair [3], possibly through its ability to interact specifically with components of the FACT chromatin remodeling complex [12], which has itself been implicated in transcriptional recovery after NER and in resistance to UV damage [33].

Accession Numbers

The ArrayExpress (https://www.ebi.ac.uk/arrayexpress) accession number for the RNA-sequencing data reported in Figure 1 is EMTAB-2754.

Supplemental Information

Supplemental Information includes Supplemental Experimental Procedures, four figures, and one table and can be found with this article online at http://dx.doi.org/10.1016/j.cub.2014.07.077.

Author Contributions

A.F. performed the experiments, analyzed the data, and wrote the paper. T.L. with A.F. performed the UV DNA fiber analysis. G.G. performed the cell-cycle analysis and global analysis of replication by DNA combing and analyzed the RNA-seq data. P.S. created the *h3.3b* line. J.E.S. conceived the study, analyzed the data, and wrote the manuscript.

Acknowledgments

The authors would like to thank Dr. Simon Elsässer (Medical Research Council Laboratory of Molecular Biology) for providing the H3.3 and H3.2 expression constructs and for comments on the manuscript, Professor Shigenori Iwai (Osaka University) for synthesizing the photoproduct-containing oligonucleotides, and members of the J.E.S. laboratory for discussions. This work was funded by the Medical Research Council through a central grant to the Laboratory of Molecular Biology (U105178808). In addition, A.F. was funded by the Jürgen Manchot Stiftung, T.L. by an EMBO Long-Term Fellowship, and G.G. by the Fanconi Anemia Research Fund.

Received: May 15, 2014
Revised: July 11, 2014
Accepted: July 30, 2014
Published: September 4, 2014

References

1. Wu, R.S., Tsai, S., and Bonner, W.M. (1982). Patterns of histone variant synthesis can distinguish G0 from G1 cells. Cell *31*, 367–374.
2. Ahmad, K., and Henikoff, S. (2002). The histone variant H3.3 marks active chromatin by replication-independent nucleosome assembly. Mol. Cell *9*, 1191–1200.
3. Adam, S., Polo, S.E., and Almouzni, G. (2013). Transcription recovery after DNA damage requires chromatin priming by the H3.3 histone chaperone HIRA. Cell *155*, 94–106.
4. Ray-Gallet, D., Quivy, J.P., Scamps, C., Martini, E.M.-D., Lipinski, M., and Almouzni, G. (2002). HIRA is critical for a nucleosome assembly pathway independent of DNA synthesis. Mol. Cell *9*, 1091–1100.
5. Ray-Gallet, D., Woolfe, A., Vassias, I., Pellentz, C., Lacoste, N., Puri, A., Schultz, D.C., Pchelintsev, N.A., Adams, P.D., Jansen, L.E.T., and Almouzni, G. (2011). Dynamics of histone H3 deposition in vivo reveal a nucleosome gap-filling mechanism for H3.3 to maintain chromatin integrity. Mol. Cell *44*, 928–941.
6. Mito, Y., Henikoff, J.G., and Henikoff, S. (2007). Histone replacement marks the boundaries of *cis*-regulatory domains. Science *315*, 1408–1411.
7. Tagami, H., Ray-Gallet, D., Almouzni, G., and Nakatani, Y. (2004). Histone H3.1 and H3.3 complexes mediate nucleosome assembly pathways dependent or independent of DNA synthesis. Cell *116*, 51–61.
8. Schneiderman, J.I., Orsi, G.A., Hughes, K.T., Loppin, B., and Ahmad, K. (2012). Nucleosome-depleted chromatin gaps recruit assembly factors for the H3.3 histone variant. Proc. Natl. Acad. Sci. USA *109*, 19721–19726.
9. Jin, C., and Felsenfeld, G. (2007). Nucleosome stability mediated by histone variants H3.3 and H2A.Z. Genes Dev. *21*, 1519–1529.
10. Lewis, P.W., Elsaesser, S.J., Noh, K.-M., Stadler, S.C., and Allis, C.D. (2010). Daxx is an H3.3-specific histone chaperone and cooperates with ATRX in replication-independent chromatin assembly at telomeres. Proc. Natl. Acad. Sci. USA *107*, 14075–14080.
11. Drané, P., Ouararhni, K., Depaux, A., Shuaib, M., and Hamiche, A. (2010). The death-associated protein DAXX is a novel histone chaperone involved in the replication-independent deposition of H3.3. Genes Dev. *24*, 1253–1265.
12. Goldberg, A.D., Banaszynski, L.A., Noh, K.-M., Lewis, P.W., Elsaesser, S.J., Stadler, S., Dewell, S., Law, M., Guo, X., Li, X., et al. (2010). Distinct factors control histone variant H3.3 localization at specific genomic regions. Cell *140*, 678–691.
13. Hödl, M., and Basler, K. (2009). Transcription in the absence of histone H3.3. Curr. Biol. *19*, 1221–1226.
14. Banaszynski, L.A., Wen, D., Dewell, S., Whitcomb, S.J., Lin, M., Diaz, N., Elsässer, S.J., Chapgier, A., Goldberg, A.D., Canaani, E., et al. (2013). Hira-dependent histone H3.3 deposition facilitates PRC2 recruitment at developmental loci in ES cells. Cell *155*, 107–120.
15. Bush, K.M., Yuen, B.T., Barrilleaux, B.L., Riggs, J.W., O'Geen, H., Cotterman, R.F., and Knoepfler, P.S. (2013). Endogenous mammalian histone H3.3 exhibits chromatin-related functions during development. Epigenetics Chromatin *6*, 7.
16. Baba, T.W., Giroir, B.P., and Humphries, E.H. (1985). Cell lines derived from avian lymphomas exhibit two distinct phenotypes. Virology *144*, 139–151.
17. Gunjan, A., and Verreault, A. (2003). A Rad53 kinase-dependent surveillance mechanism that regulates histone protein levels in *S. cerevisiae*. Cell *115*, 537–549.
18. Hake, S.B., Garcia, B.A., Kauer, M., Baker, S.P., Shabanowitz, J., Hunt, D.F., and Allis, C.D. (2005). Serine 31 phosphorylation of histone variant H3.3 is specific to regions bordering centromeres in metaphase chromosomes. Proc. Natl. Acad. Sci. USA *102*, 6344–6349.
19. Schwartzentruber, J., Korshunov, A., Liu, X.-Y., Jones, D.T.W., Pfaff, E., Jacob, K., Sturm, D., Fontebasso, A.M., Quang, D.-A.K., Tönjes, M., et al. (2012). Driver mutations in histone H3.3 and chromatin remodelling genes in paediatric glioblastoma. Nature *482*, 226–231.
20. Wu, G., Broniscer, A., McEachron, T.A., Lu, C., Paugh, B.S., Becksfort, J., Qu, C., Ding, L., Huether, R., Parker, M., et al.; St. Jude Children's Research Hospital–Washington University Pediatric Cancer Genome Project (2012). Somatic histone H3 alterations in pediatric diffuse intrinsic pontine gliomas and non-brainstem glioblastomas. Nat. Genet. *44*, 251–253.
21. Behjati, S., Tarpey, P.S., Presneau, N., Scheipl, S., Pillay, N., Van Loo, P., Wedge, D.C., Cooke, S.L., Gundem, G., Davies, H., et al. (2013). Distinct H3F3A and H3F3B driver mutations define chondroblastoma and giant cell tumor of bone. Nat. Genet. *45*, 1479–1482.
22. Bjerke, L., Mackay, A., Nandhabalan, M., Burford, A., Jury, A., Popov, S., Bax, D.A., Carvalho, D., Taylor, K.R., Vinci, M., et al. (2013). Histone H3.3 mutations drive pediatric glioblastoma through upregulation of MYCN. Cancer Discov. *3*, 512–519.
23. Lewis, P.W., Müller, M.M., Koletsky, M.S., Cordero, F., Lin, S., Banaszynski, L.A., Garcia, B.A., Muir, T.W., Becher, O.J., and Allis, C.D. (2013). Inhibition of PRC2 activity by a gain-of-function H3 mutation found in pediatric glioblastoma. Science *340*, 857–861.
24. Mejlvang, J., Feng, Y., Alabert, C., Neelsen, K.J., Jasencakova, Z., Zhao, X., Lees, M., Sandelin, A., Pasero, P., Lopes, M., and Groth, A. (2014). New histone supply regulates replication fork speed and PCNA unloading. J. Cell Biol. *204*, 29–43.
25. Edmunds, C.E., Simpson, L.J., and Sale, J.E. (2008). PCNA ubiquitination and REV1 define temporally distinct mechanisms for controlling translesion synthesis in the avian cell line DT40. Mol. Cell *30*, 519–529.
26. Jansen, J.G., Tsaalbi-Shtylik, A., Hendriks, G., Gali, H., Hendel, A., Johansson, F., Erixon, K., Livneh, Z., Mullenders, L.H.F., Haracska, L., and de Wind, N. (2009). Separate domains of Rev1 mediate two modes of DNA damage bypass in mammalian cells. Mol. Cell. Biol. *29*, 3113–3123.
27. Szüts, D., Marcus, A.P., Himoto, M., Iwai, S., and Sale, J.E. (2008). REV1 restrains DNA polymerase ζ to ensure frame fidelity during translesion synthesis of UV photoproducts in vivo. Nucleic Acids Res. *36*, 6767–6780.
28. Law, M.J., Lower, K.M., Voon, H.P.J., Hughes, J.R., Garrick, D., Viprakasit, V., Mitson, M., De Gobbi, M., Marra, M., Morris, A., et al. (2010). ATR-X syndrome protein targets tandem repeats and influences allele-specific expression in a size-dependent manner. Cell *143*, 367–378.
29. Clynes, D., Jelinska, C., Xella, B., Ayyub, H., Taylor, S., Mitson, M., Bachrati, C.Z., Higgs, D.R., and Gibbons, R.J. (2014). ATRX dysfunction induces replication defects in primary mouse cells. PLoS ONE *9*, e92915.
30. Sale, J.E. (2012). Competition, collaboration and coordination—determining how cells bypass DNA damage. J. Cell Sci. *125*, 1633–1643.
31. Sale, J.E. (2013). Translesion DNA synthesis and mutagenesis in eukaryotes. Cold Spring Harb. Perspect. Biol. *5*, a012708.
32. Lin, J.-R., Zeman, M.K., Chen, J.-Y., Yee, M.-C., and Cimprich, K.A. (2011). SHPRH and HLTF act in a damage-specific manner to coordinate different forms of postreplication repair and prevent mutagenesis. Mol. Cell *42*, 237–249.
33. Dinant, C., Ampatziadis-Michailidis, G., Lans, H., Tresini, M., Lagarou, A., Grosbart, M., Theil, A.F., van Cappellen, W.A., Kimura, H., Bartek, J., et al. (2013). Enhanced chromatin dynamics by FACT promotes transcriptional restart after UV-induced DNA damage. Mol. Cell *51*, 469–479.

Current Biology, Volume *24*

Supplemental Information

Histone H3.3 Is Required to Maintain Replication Fork Progression after UV Damage

Alexander Frey, Tamar Listovsky, Guillaume Guilbaud, Peter Sarkies, and Julian E. Sale

Figure S1

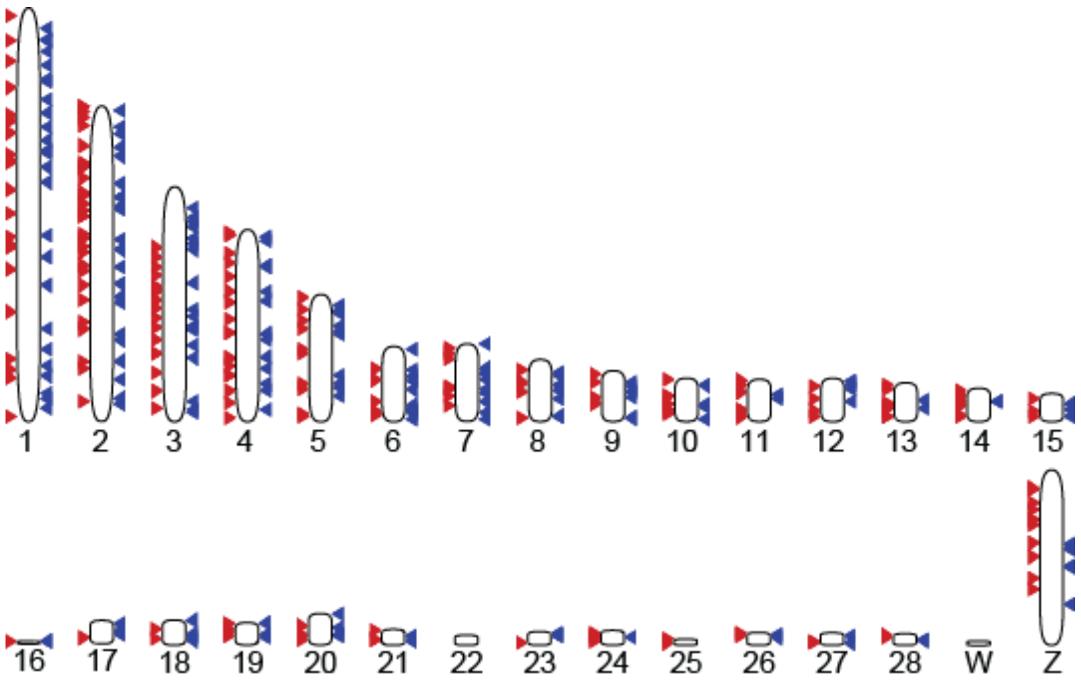

Figure S1. (Related to Figure 1). Dysregulated genes (Table S1) mapped to the genome. Genes with increased expression in *h3.3*, red; genes with decreased expression in *h3.3*, blue.

Figure S2

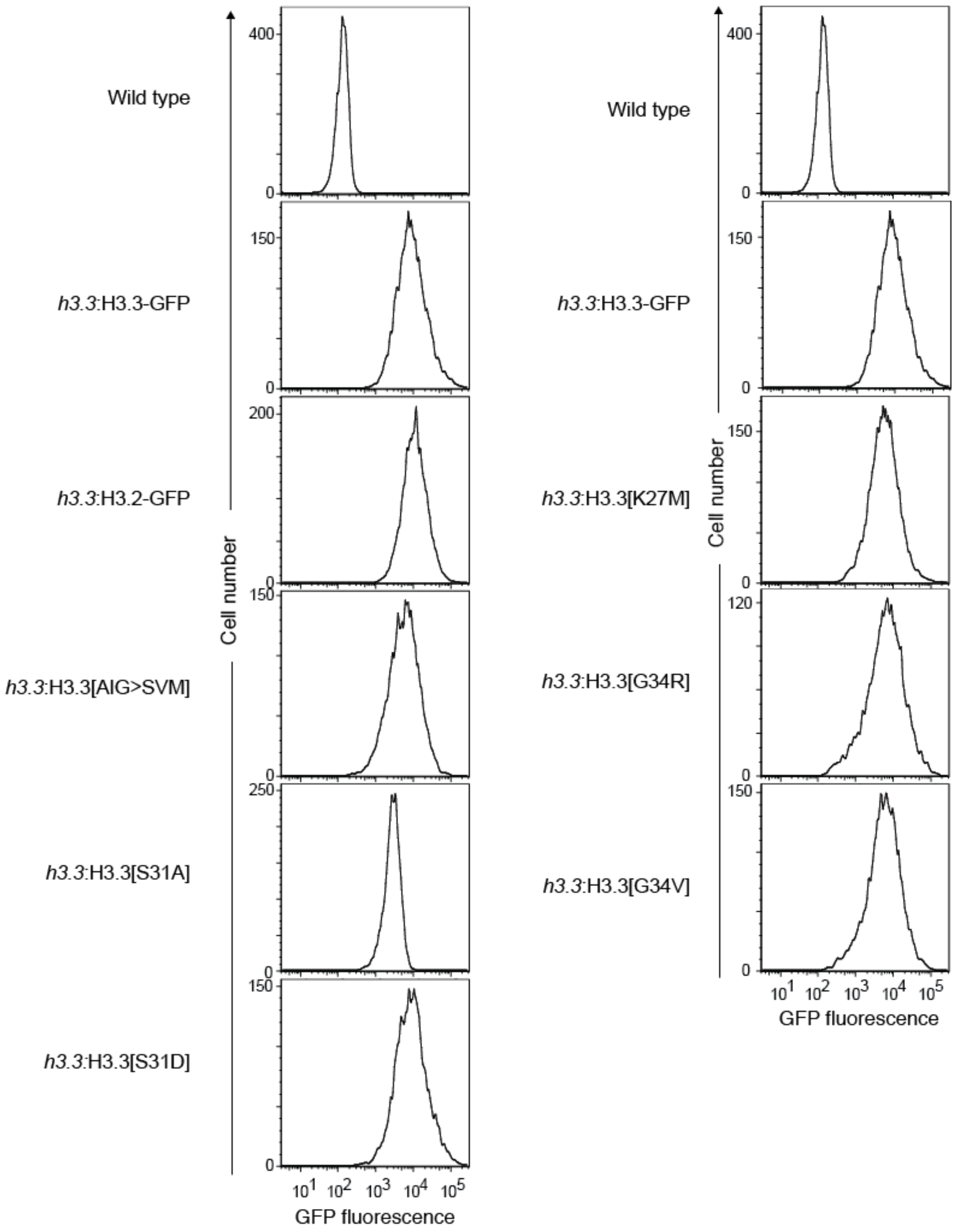

Figure S2. (Related to Figure 2). Flow cytometry for GFP was used to match expression of GFP-histone fusions expressed in the complemented *h3.3* lines used in this study.

Figure S3

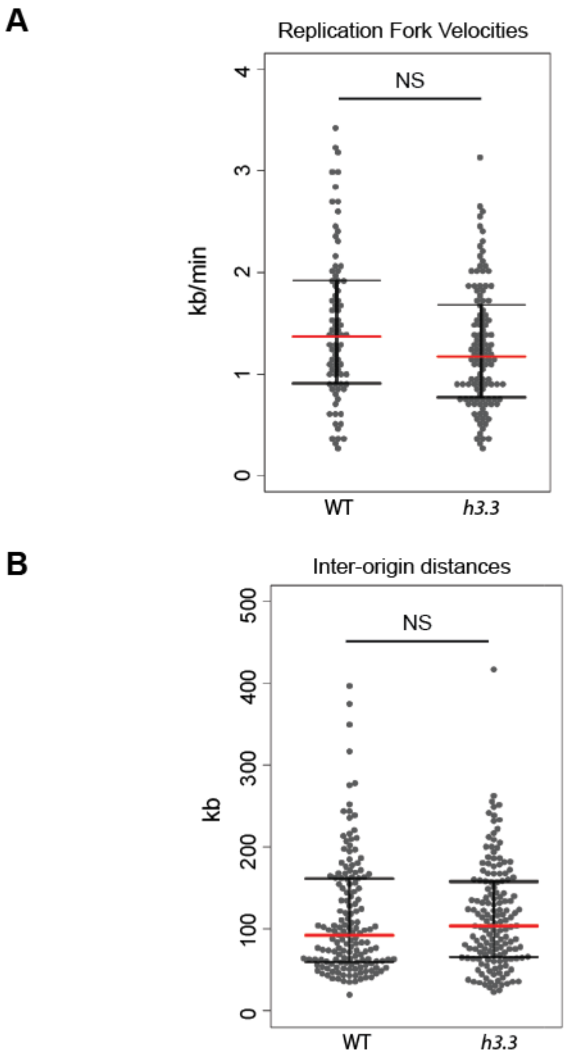

C

	WT	*h3.3*
Mean fibre length (kb)	701 ± 51 (n=65)	535 ± 44 (n=73)
Total DNA analysed (Mb)	38.6	38.2
Origin density (per Mb)	5.4	5.8
Inter-origin distance (kb)	114 ± 6 (n=156)	115 ± 5 (n=156)
Fork velocity (kb/min)	1.48 ± 0.09 (n=79)	1.25 ± 0.06 (n=114)

Figure S3. (Related to Figure 3). A – C. Unperturbed replication dynamics in *h3.3* cells monitored in DNA counterstained fibres (see Supplemental Experimental Procedures). Only the first labelling period (IdU) is measured, as described in reference [S1]. A. Replication fork velocities. B Interorigin distances. In A and B the red line represents the median and whiskers = interquartile range. Mann-Whitney test: p = not significant (NS; $p > 0.05$). C. Summary analysis. Values are presented as mean +/- SEM.

Figure S3 (continued)

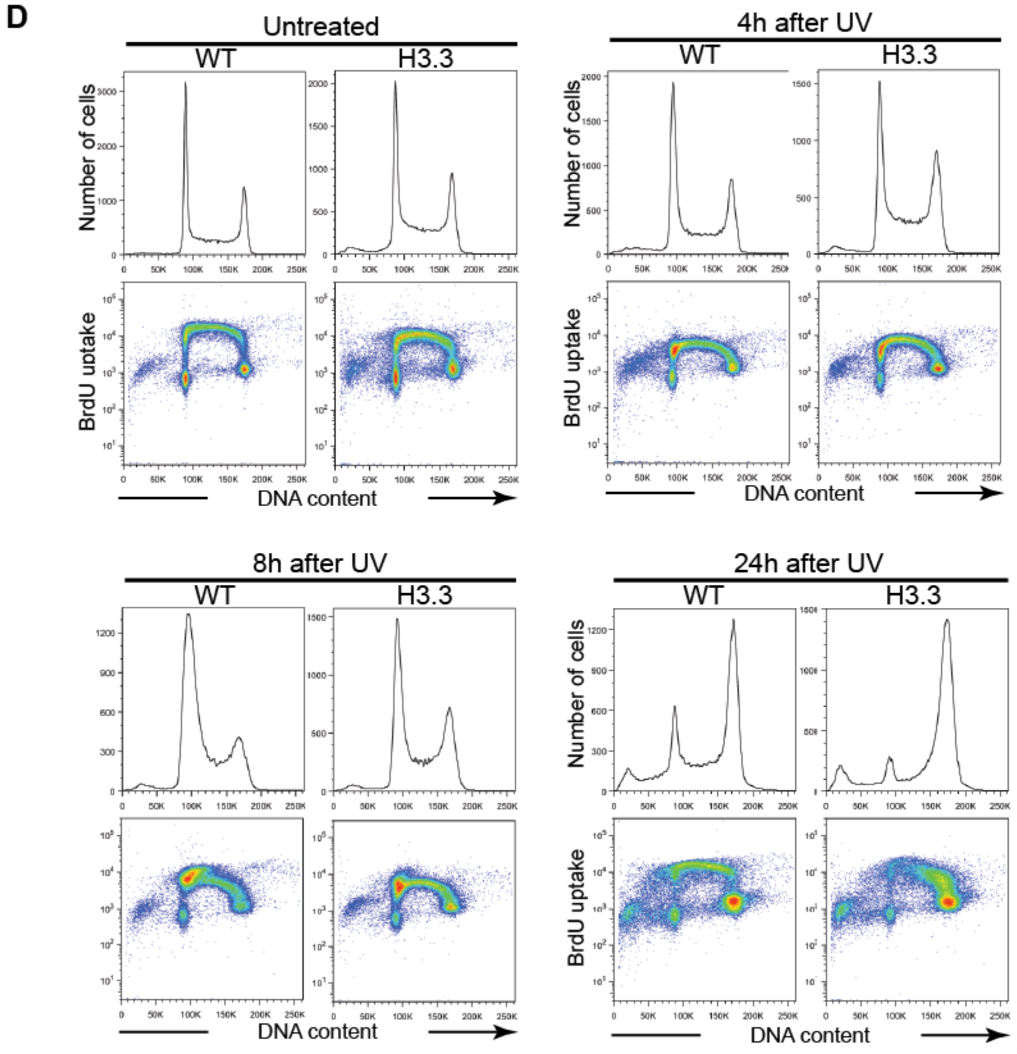

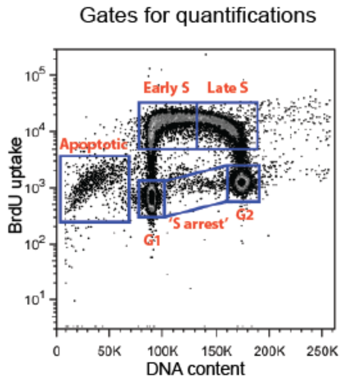

Time after UV

% of cycling cells	Mock WT	Mock *H3.3*	4h WT	4h *H3.3*	8h WT	8h *H3.3*	24h WT	24h *H3.3*
G1	21	17	10	6	6	7	8	4
Early S	38	41	46	46	65	51	25	10
Late S	20	20	22	23	18	23	17	34
G2	19	18	19	22	12	17	44	38
S arrest	1	2	1	1	1	1	4	2
Apoptotic	2	6	7	5	3	4	12	12

Figure S3 (continued). (Related to Figure 3). D. Cell cycle response of wild type and *h3.3* cells to UV irradiation. 1D and 2D cell cycle analysis of asynchronous populations of wild type and *h3.3* cells before and after exposure to 3 Jm^{-2} UVC irradiation. A key for the gating is shown and a table for the percentage of counts in each gate for each condition.

Figure S3 (continued)

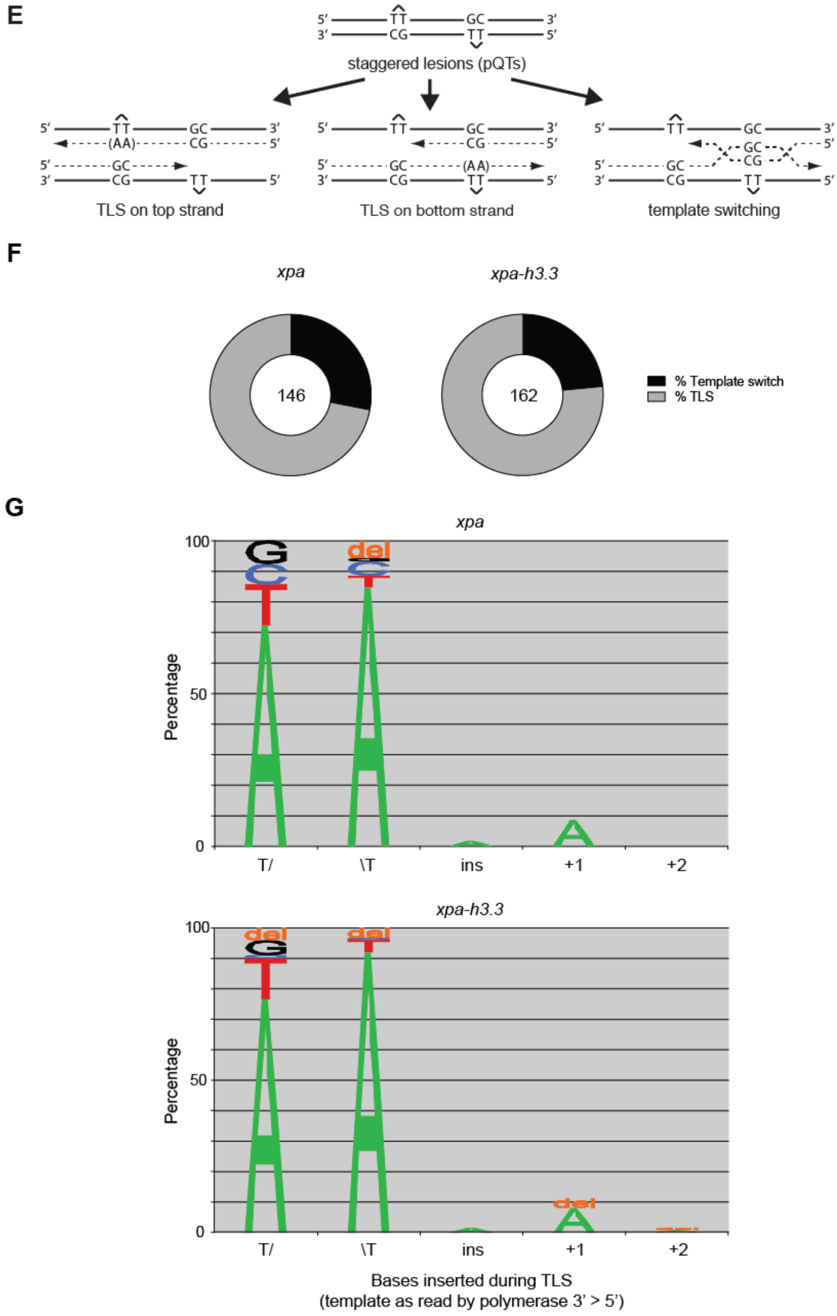

Figure S3 (continued). (Related to Figure 3). Bypass of (6-4)-photoproducts in *h3.3* cells. E. Schematic of the staggered photoproducts in the replicating plasmid pQ1 and the possible outcomes of their replication [taken from S2]. F. Percentage of lesions replicated by presumed 'template switching' vs. translesion synthesis (TLS). The number in the centre of the pie chart indicates the number of analysed sequences. G. Representation of the bases inserted during TLS. The order follows the template as read by the polymerase, 3' T of lesion (T/), 5' T of lesion (/T), non-templated insertion (extra base), mutation at the +1 and +2 positions [see also S2]. *xpa* is used as a control to eliminate any contribution of excision repair, which would give the same outcome as template switching.

Figure S4

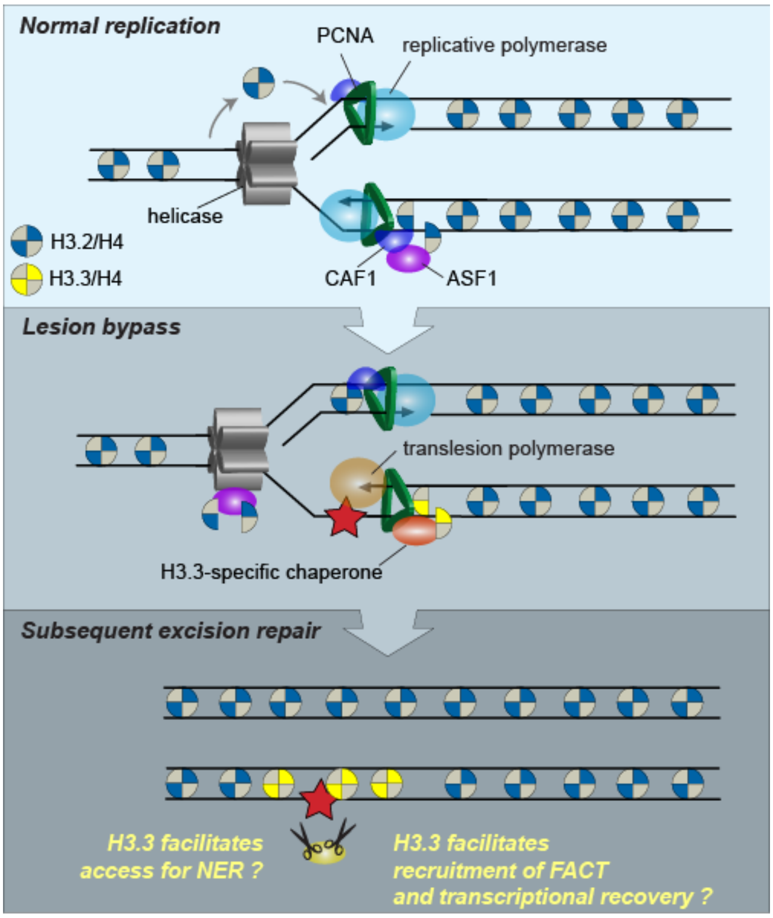

Figure S4. A theoretical model for the role of H3.3 during DNA damage bypass and excision repair. During normal replication both recycled H3/H4 and newly synthesised H3.2/H4 are incorporated into the nascent daughter strands, chaperones by CAF1 and ASF1 (Top panel). When the fork encounters a lesion (Red star, middle panel) we propose that the histone chaperone switches to one of the H3.3-specific chaperones (for instance HIRA or ATRX/DAXX). The absence of H3.3 in these circumstances causes a delay in fork progression. We suggest that, under normal circumstances, H3.3 is incorporated as the fork bypasses the lesion preventing any delay and marking the site of lesion bypass with H3.3. We propose that the presence of H3.3 either improves access for the NER apparatus and / or facilitates transcriptional recovery after repair (Lower panel).

Table S1. List of genes significantly up or down-regulated in *h3.3* vs. wildtype RNA-seq. (Related to Figure 1). Separate Excel spreadsheet listing the genes upregulated and downregulated more than 2-fold with a p value of < 0.001. The complete RNA-seq datasets (three each of wild type and *h3.3*) have been deposited in Array Express (https://www.ebi.ac.uk/arrayexpress/) with accession number E-MTAB-2754.

Supplemental Experimental Procedures

DT40 cell culture & complementation

Wild type DT40 and its derivatives were cultured as previously described [S3]. Growth was monitored with a Vi-Cell counter (Beckman-Coulter). Generation of lines stably expressing H3.3-GFP was performed as described [S4]. Briefly, 2 x10^7 cells were transfected with 20 μg plasmid DNA (see 'H3.3 constructs and site directed mutagenesis' below) in a BioRad Gene Pulser with 0.4mm cuvettes at 250 V, 950 μF. Drug resistant clones were expanded and assessed for GFP expression. Clones were selected to have matched GFP expression (Figure S2).

Gene targeting

H3.3B

To delete the H3.3B gene we created a targeting construct by amplifying a genomic region including the entire H3.3B gene with primers H33BF1 and H33BR1. The PCR product was cloned into pBluescript and the EcoRV fragment that contains most of the coding region was replaced with a selection cassette (blasticidin/puromycin) by blunt ligation. Drug-resistant clones were screened for targeted integration by digestion of genomic DNA with NcoI followed by Southern blotting with a probe 5' of the targeting construct generated with the primers H3.3B-Probe-F and H3.3B-Probe-R. Two homozygous H3.$3^{-/-}$ clones were obtained, one of which was taken forward for targeting of the *H3.3A* locus.

H3.3A

To disrupt the H3.3A gene we created a targeting construct by amplifying genomic regions up- and downstream of the H3.3A coding region with primers H33AF1 and H33AR1, H33AF2 and H33AR2. The 5' arm was cloned into TOPO and moved to pBluescript as an ApaI fragment. The PCR product of the 3' arm was digested with SacI and cloned into pBluescript. A selection cassette (conferring blasticidin resistance) was inserted to replace the entire coding region of H3.3A. Drug-resistant clones were screened for targeted integration by PCR with the primers H33A-1-F and H33A-1-R for the first allele, and H33A-2-F and H33A-2-R for the second allele. We obtained two *h3.3* lines, c20 and c32. Both behaved identically. c20 was used for most studies except where stated.

XPA
To delete the XPA gene we used a previously described puromycin resistant targeting construct [S5]. Clones were screened for targeted integration by PCR with the primers XPAF and XPAR.

Colony survival assays
Colony survival experiments were carried out in methylcellulose medium as previously described [S6]. UV light at 254 nm was delivered using a custom-made shuttered source and calibrated with a UV radiometer (UVP, Upland, CA 91786, USA). Cisplatin (CDDP) and methyl methanesulphonate were obtained from Sigma. The D_{10} (dose resulting in a 10% survival) was calculated for each curve and the fold sensitivity of each mutant relative to wild type is given in the relevant figure legend.

H3 constructs and site directed mutagenesis
The pCDH expression vectors which express H3.2 and H3.3 with GFP fused to the C-terminus [S7] were kindly provided by Dr. Simon Elsässer. Site directed mutagenesis of H3.3 was performed as previously described [S8]. Primers used to generate the H3.3[AIG>SVM], H3.3[S31A], H3.3[S31D], H3.3[K27M], H3.3[G34R] and H3.3[G34V] constructs are listed below.

RNA deep sequencing for gene expression analysis
Three wild type and three *h3.3* DT40 pools were expanded for 3 weeks after which RNA was extracted using an RNeasy Mini Kit (Qiagen). The RNA quality and quantity was determined using an Agilent 2100 Bioanalyzer (Agilent Technologies). Sequencing of RNA from each pool was carried out by BGI (Beijing Genomics Institute) using an Illumina HiSeq2000. FASTQ files were aligned to the Chicken cDNA derived from the Galgal4 genome assembly (from:ftp://ftp.ensembl.org/pub/release-74/fasta/gallus_gallus/cdna/) using Bowtie [S9]. A maximum of two mismatches per read were allowed. Only sequences that mapped to one location on the genome were retained. Read counts were identified for each mapped transcript. The DEseq package [S10] from Bioconductor (http://bioconductor.org/) was then used to normalise read counts. Differential expression and associated statistical significance was computed using a negative binomial test from the DESeq package. Graphical representations and further analysis were performed using homemade R scripts. The mapping of dysregulated genes to the chicken karyotype was performed using Ensembl (http://www.ensembl.org/index.html).

Western blotting
Cells were lysed in extraction buffer (50 mM Tris-HCl, pH 8, 250 mM NaCl, 20 mM EGTA, 50 mM NaF, and 1% Triton X-100) and Complete Protease Inhibitor Cocktail (Roche) on ice for 20 minutes. Lysates were cleared by centrifugation at 13’000 rpm for 20 min at 4°C. Extracts were boiled in Laemmli buffer for 5 minutes. Protein

levels were quantified before loading on NuPAGE gels (Life Technologies) and transferred on nitrocellulose membranes (Whatman). Antibodies used at a 1/1000 dilution: anti-Histone H3 (Abcam, ab1791), anti-Histone H3.3 (EMD Millipore, 09-838). Antibodies used at a 1/2000 dilution: anti-mouse (Jackson ImmunoResearch, 115-035-174), anti-rabbit (Jackson ImmunoResearch, 211-032-171).

Flow cytometry

Flow cytometry to assess GFP expression was performed using an LSRII cytometer (BD Biosciences).

Flow-cytometric analysis of cell-cycle progression

In order to monitor the cell cycle after UV treatment (3 J/m^2), cells were incubated 30 minutes before the indicated time with 50μM BrdU. Cells were then placed in 3 volumes of ice cold 1X PBS, spun down at 400g, washed once in cold 1X PBS and then fixed in 75% EtOH for a minimum of 24 hours at -20ºC. Each sample was then spun down and incubated in 15 mM pepsin/30 mM HCl for 20 min at 37°C. The DNA was denatured in 2M HCl for 20 min at room temperature. Cells were then washed once in 1X PBS and resuspended in antibody dilution buffer (0.75% FCS, 0.25% chicken serum, 0.5% Tween 20, 20 mM HEPES in PBS). The pellets were then sequentially incubated for 1h in 1/5 mouse anti-BrdU (Becton Dickinson), 30 minutes 1/50 rabbit anti-mouse Alexa-Fluor 594 (Life Technologies), and 30 minutes 1/50 donkey anti-rabbit Alexa-Fluor 594 (Life Technologies). Total DNA was stained in 1μM Hoechst 33342. Analyses were carried out on a LSRII (BD Sciences). 50,000 unique cells were counted for each sample.

Preparation, spreading and immunolabelling of DNA Fibres

This method is essentially that used in [S11], but with modifications. Exponentially growing DT40 cells (6 x10^6) were incubated at 37 °C with 50 µM IdU for 20 min. They were then spun down and incubated at 37 °C with 50 µM CldU for 20 min. For UV treatment, after labelling with IdU cells were irradiated with 40 J/m^2 254 nm light in 1 ml of PBS and then incubated with CldU as above. Labelled cells were resuspended in PBS to a concentration of 1×10^6 cells ml^{-1}. Three μl were spotted onto clean glass Superfrost slides and lysed with 7 µl of 0.5% SDS in 200 mM Tris-HCl (pH 5.5) and 50 mM EDTA (5 min, 20°C). Slides were tilted at 15° to horizontal, allowing the DNA to run slowly down the slide. Slides were then air dried and fixed in 3:1 methanol/acetic acid, and stored at 4°C before immunolabelling.

The DNA fibre spreads were hydrated with water and then denatured with 2.5M HCl for 1hr at 20°C. Slides were washed three times in PBS, then incubated in PBS containing 1% BSA and 0.1% Tween 20 for 1 hr at 20°C. Slides were incubated (45 min, 20 °C) with rat anti-BrdU (Oxford Biotechnology Ltd.) at 1:500 to detect CldU. Slides were then washed three times in PBS and incubated (20 min, 20 °C) with AF488 chicken anti-rat antibody (Invitrogen, Life Technologies) at 1:100. Slides were washed three times in PBS and incubated (20 min, 20°C) with AF488 goat anti-

chicken antibody (Invitrogen, Life Technologies) at 1:100. The slides were then again washed three times with PBS and incubated (45 min, 20°C) with mouse anti-BrdU (BD Biosciences) at 1:10 to detect IdU. Slides were washed three times in PBS and incubated (20 min, 20°C) with AF594 rat anti-mouse antibody (Invitrogen, Life Technologies) at 1:100. Slides were washed three times in PBS and incubated (20 min, 20°C) with AF594 donkey anti-rat antibody (Invitrogen, Life Technologies) at 1:100. Finally, slides were washed three times in PBS and mounted in Fluoromount G (Southern Biotechnology). Slides were kept at 4°C and imaged using a Nikon C1-si confocal microscope. Tract lengths were measured using Adobe Photoshop.

For analysis of DNA replication dynamics (fork velocity and origin density) we additionally revealed the DNA with an anti-DNA antibody as previously described [S1].

Replicating plasmid assay
The replicating plasmid assay was carried out as previously described [2]. The 6-4 photoproduct in a staggered configuration was used in pQ1. The lesion containing oligos were provided by Professor Shigenori Iwai, Osaka University.

Oligonucleotides

Name	Sequence (5' to 3')
H33AF1	CCCTCTGTTGGATGTAGGACA
H33AR1	CCGTGGACTTCATTTAGAGCA
H33AF2	TGGGTAGAGTCTGGAGCTGAA
H33AR2	CCTCTTGGTGTGAAGCAGAAC
H33A-1-F	TGGTTTGTCCAAACTCATCAA
H33A-1-R	CACAGTGCCATTTGGGTTTA
H33A-2-F	AAGGGCCTTCTCTCTGTTAGC
H33A-2-R	CACAGTGCCATTTGGGTTTA
H33BF1	ACCTCAGGGCAGGTGACACAAAACC
H33BR1	GGTGTTCTACTGATGGAAAGGGGAG
H3.3B-Probe-F	CTACTGATGGAAAGGGGAGATAGG
H3.3B-Probe-R	TAAGCCTAAGCTGGTGTCCTGAGAG
XPAF	GGTGGGGCTGATAGTGTGTAA
XPAR	GATGGAGGAACGAACTGACAA
K27MF	GCCGCCCGCATGAGCGCCCCG
K27MR	CGGGGCGCTCATGCGGGCGGC
S31AF	GAGCGCCCCGGCCACCGGCGG
S31AR	CCGCCGGTGGCCGGGGCGCTC
S31DF	CAAGAGCGCCCCGGACACCGGCGGCGTG
S31DR	CACGCCGCCGGTGTCCGGGGCGCTCTTG
G34RF	CCGTCCACCGGCAGGGTGAAGAAGCCTC
G34RR	GAGGCTTCTTCACCCTGCCGGTGGACGG

G34VF	CCGTCCACCGGCGTGGTGAAGAAGCCTC
G34VR	GAGGCTTCTTCACCACGCCGGTGGACGG
AIGF	CTCCTGCAGAGCCATGACGGCCGAGCTCTGGAAGCGC
AIGR	GCGCTTCCAGAGCTCGGCCGTCATGGCTCTGCAGGAG

Supplemental References

S1. Guilbaud, G., Rappailles, A., Baker, A., Chen, C.-L., Arneodo, A., Goldar, A., d'Aubenton-Carafa, Y., Thermes, C., Audit, B., and Hyrien, O. (2011). Evidence for sequential and increasing activation of replication origins along replication timing gradients in the human genome. PLoS Comput. Biol. *7*, e1002322.

S2. Szüts, D., Marcus, A. P., Himoto, M., Iwai, S., and Sale, J. E. (2008). REV1 restrains DNA polymerase zeta to ensure frame fidelity during translesion synthesis of UV photoproducts in vivo. Nucleic Acids Res. *36*, 6767–6780.

S3. Simpson, L. J., and Sale, J. E. (2003). Rev1 is essential for DNA damage tolerance and non-templated immunoglobulin gene mutation in a vertebrate cell line. EMBO J. *22*, 1654–1664.

S4. Sale, J. E. (2006). Stable non-targeted transfection of DT40. Subcell. Biochem. *40*, 341–344.

S5. Okada, T., Sonoda, E., Yamashita, Y. M., Koyoshi, S., Tateishi, S., Yamaizumi, M., Takata, M., Ogawa, O., and Takeda, S. (2002). Involvement of vertebrate polkappa in Rad18-independent postreplication repair of UV damage. J. Biol. Chem. *277*, 48690–48695.

S6. Simpson, L. J., and Sale, J. E. (2006). Colony survival assay. Subcell. Biochem. *40*, 387–391.

S7. Elsässer, S. J., Huang, H., Lewis, P. W., Chin, J. W., Allis, C. D., and Patel, D. J. (2012). DAXX envelops a histone H3.3-H4 dimer for H3.3-specific recognition. Nature *491*, 560–565.

S8. Ross, A.-L., Simpson, L. J., and Sale, J. E. (2005). Vertebrate DNA damage tolerance requires the C-terminus but not BRCT or transferase domains of REV1. Nucleic Acids Res. *33*, 1280–1289.

S9. Langmead, B., Trapnell, C., Pop, M., and Salzberg, S. L. (2009). Ultrafast and memory-efficient alignment of short DNA sequences to the human genome. Genome Biol. *10*, R25.

S10. Anders, S., and Huber, W. (2010). Differential expression analysis for sequence count data. Genome Biol. *11*, R106.

S11. Edmunds, C. E., Simpson, L. J., and Sale, J. E. (2008). PCNA ubiquitination and REV1 define temporally distinct mechanisms for controlling translesion synthesis in the avian cell line DT40. Molecular Cell *30*, 519–529.
